# Programmed cell death in degenerative skeletal diseases: molecular crosstalk and combinatorial therapeutic strategies

**DOI:** 10.3389/fcell.2026.1876124

**Published:** 2026-09-04

**Authors:** Rui Zhao, Jirong Zhao, Hongnian Yin, Yang Zhan, Dong Ma, Xu Xue, Wentong Yang

**Affiliations:** Clinical College of Chinese Medicine, Gansu University of Chinese Medicine, Lanzhou, China

**Keywords:** intervertebral disc degeneration, molecular crosstalk, osteoarthritis, osteoporosis, programmed cell death, translational barriers

## Abstract

Degenerative skeletal diseases, including osteoarthritis (OA), intervertebral disc degeneration (IVDD) and osteoporosis (OP), are major causes of chronic pain, disability and loss of skeletal function, yet effective disease-modifying therapies remain limited. Although these disorders affect distinct tissues and cell populations, they share persistent inflammation, oxidative stress, mitochondrial dysfunction, metabolic imbalance and defective tissue remodelling. Programmed cell death (PCD) is a critical link between these disturbances and progressive structural failure. Apoptosis, pyroptosis, necroptosis, ferroptosis and autophagy-associated cell-fate regulation rarely operate independently. Their activation and biological consequences are determined by cell type, anatomical niche, disease stage and stress intensity. However, the concurrent detection of multiple death-associated markers is often misinterpreted as mechanistic crosstalk, hindering accurate identification of causal pathways and therapeutic targets. This Review proposes an evidence-graded, disease-stage-, cell-type- and microenvironment-dependent framework for interpreting PCD interactions in OA, IVDD and OP. It distinguishes shared upstream triggers, convergent regulatory nodes, sequential transitions, direct molecular conversion and functional coexistence according to their evidential strength. We further evaluate therapeutic strategies targeting the cell death–inflammation–metabolism axis, including multi-target agents, nanomaterials, extracellular vesicles, biomaterials and tissue engineering. Most evidence remains preclinical, underscoring the urgent need for human validation, causal target confirmation and rigorous assessment of delivery, durability, safety and manufacturability. This framework may improve mechanistic precision and accelerate the development of context-specific therapies for degenerative skeletal diseases.

## 1 Introduction

Increasing evidence implicates multiple regulated cell-death pathways and autophagy-associated stress responses in the progression of degenerative skeletal diseases. Osteoarthritis (OA), intervertebral disc degeneration (IVDD) and osteoporosis (OP) are clinically, anatomically and biologically distinct skeletal disorders. OA is a progressive disease of synovial joints involving multiple tissues, including articular cartilage, synovium, subchondral bone, ligaments and other periarticular structures (Lane et al., 2011). IVDD affects the nucleus pulposus, annulus fibrosus and cartilaginous endplates within a mechanically constrained and poorly vascularized environment (Fardon et al., 2014). OP is characterized by compromised bone strength resulting from reduced bone mass, deterioration of skeletal microarchitecture and increased fracture susceptibility (NIH Consensus Development Panel on Osteoporosis Prevention, Diagnosis, and Therapy, 2001). Experimental disease-modifying approaches, including extracellular-vesicle-based strategies, have shown efficacy in animal models of OA (Duan et al., 2021), but broad clinical translation remains limited. Across OA, IVDD and OP, progressive structural failure and the loss or dysfunction of tissue-maintaining cells remain major contributors to pain, disability and impaired skeletal function.

Despite their substantial differences, these diseases can be considered within a shared conceptual framework because they exhibit recurrent pathological disturbances, including inflammation, oxidative and mitochondrial stress, metabolic dysregulation and impaired extracellular-matrix or bone-remodelling homeostasis. Representative studies have shown that these disturbances can alter the survival and function of chondrocytes, osteoblasts, osteocytes and osteoclast-lineage cells through apoptosis, pyroptosis, ferroptosis and autophagy-associated responses (Li et al., 2023a; Shi et al., 2024; Tao et al., 2024; Wan et al., 2021; Gao W. et al., 2024; Wang S. et al., 2022; Behera et al., 2022). However, shared upstream disturbances do not imply that the same death pathway has equivalent functions in different diseases, anatomical compartments or cell populations.

Experimental studies have linked mechanical overloading, mitochondrial DNA release, ferritinophagy and disturbed redox homeostasis to ferroptotic or pyroptotic injury in disease-specific skeletal cells (Wang S. et al., 2022; Zhang W. et al., 2022; Sun et al., 2023a; Jiang Z. et al., 2024). These observations indicate that different cell-death-associated responses may be activated in parallel or regulated by common upstream stress pathways. More restricted forms of direct interaction may occur at the level of selective-autophagy cargo or cell-death execution machinery. For example, activation of the JNK–jun proto-oncogene, AP-1 transcription factor subunit (JUN)–nuclear receptor coactivator 4(NCOA4) axis promotes ferritinophagy, increases intracellular iron availability and enhances chondrocyte ferroptosis (Sun et al., 2023a). By contrast, representative OA studies indicate that TFEB-dependent autophagy and FUNDC1-dependent mitophagy act predominantly as adaptive quality-control mechanisms that limit endoplasmic-reticulum stress, mitochondrial dysfunction and cartilage injury (Wu et al., 2023; Fang et al., 2023). Such context-dependent regulation may contribute to variable responses to single-target interventions, although direct comparisons between single- and multi-pathway approaches remain limited.

A major interpretive problem is that concurrent detection or simultaneous suppression of several death-associated markers is often described as molecular crosstalk. Such findings may instead reflect regulation by a common upstream stressor. For example, modulation of the MYD88–NF-κB axis alters both apoptotic and pyroptotic readouts in experimental OA but does not demonstrate direct interaction between their execution machineries (Chen J. et al., 2023). Mechanistic studies outside degenerative skeletal disease have shown that caspase-3-mediated cleavage of gasdermin E (GSDME) can convert an initially apoptotic response into a lytic, pyroptosis-like phenotype (Wang et al., 2017; Rogers et al., 2017). Similar precision is required when interpreting autophagy and pyroptosis, apoptosis and necroptosis (PANoptosis). Most skeletal-disease studies describe altered autophagic flux, mitophagy or selective autophagy as adaptive or cell-fate-modifying processes rather than *bona fide* autophagy-dependent cell death (Wu et al., 2023; Fang et al., 2023). Autosis provides an experimentally defined example of cell death that is dependent on the autophagic machinery and regulated by Na+,K + -ATPase (Liu et al., 2013); however, broader claims of autophagy-dependent cell death should require pathway-specific evidence that disruption of the essential autophagic machinery prevents cell demise. Likewise, PANoptosis should not be inferred solely from simultaneous changes in apoptotic, pyroptotic and necroptotic markers. Mechanistic studies in infectious models have demonstrated coordinated activation of these execution branches and the formation of ZBP1- or AIM2-associated PANoptosome complexes (Banoth et al., 2020; Lee et al., 2021). Accordingly, claims of PANoptosis in degenerative skeletal diseases should require evidence of coordinated pathway dependency, integrated execution and PANoptosome-related molecular organization rather than marker co-expression alone.

The distinctive contribution of this critical narrative Review is therefore not the addition of further cell-death categories, but the development of a disease-stage-, cell-type- and microenvironment-dependent framework for interpreting cell-death relationships in OA, IVDD and OP. Reported interactions are classified as shared upstream triggers, shared regulatory nodes, functional coexistence, sequential transitions or direct molecular conversion, each of which requires a different level of evidence. Their pathological relevance is evaluated according to the affected cell population, anatomical niche, disease stage and surrounding inflammatory, redox, metabolic and mechanical environment. Evidence from cellular systems, animal models, *ex vivo* tissues, human specimens and, where available, clinical studies is considered separately to avoid drawing equivalent causal conclusions from unequal experimental evidence. This framework aims to distinguish genuine mechanistic crosstalk from parallel pathway activation and to identify the disease contexts in which multi-pathway intervention may be biologically and translationally justified.

## 2 Principal regulated cell-death programmes and associated stress responses

**FIGURE 1:**
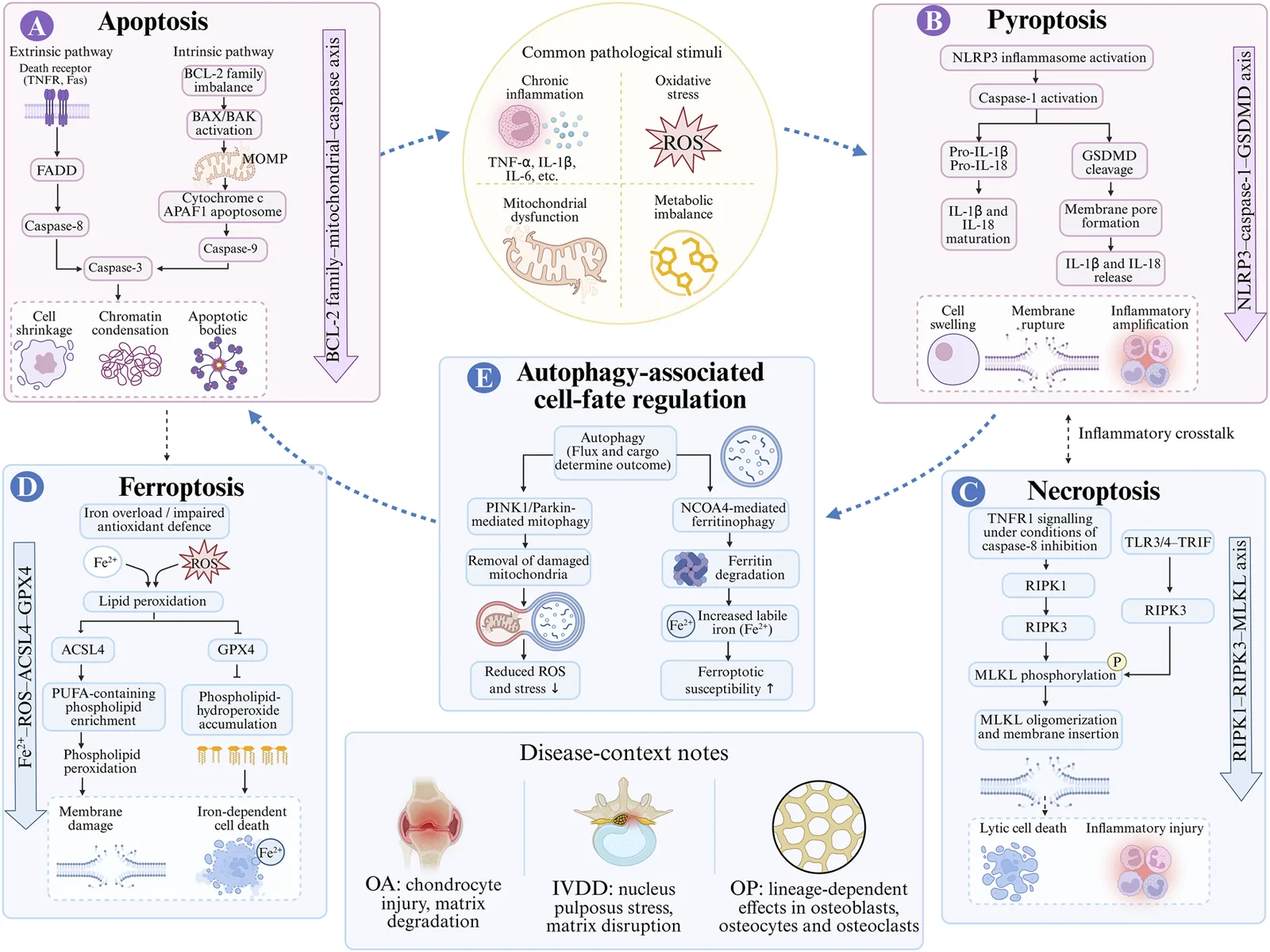
Principal regulated cell-death programmes and associated stress responses in degenerative skeletal diseases. Common pathological stimuli, including chronic inflammation, oxidative stress, mitochondrial dysfunction and metabolic imbalance, modify the susceptibility of skeletal cells to distinct cell-death programmes. **(A)** Apoptosis is initiated through extrinsic death-receptor signalling or intrinsic BCL-2-family-regulated mitochondrial outer-membrane permeabilization, with both pathways converging on caspase-3 activation. **(B)** Canonical pyroptosis involves NLRP3-inflammasome-dependent caspase-1 activation, cytokine maturation and GSDMD-mediated membrane-pore formation. **(C)** Necroptosis is mediated by RIPK1–RIPK3–MLKL signalling downstream of TNFR1 or TLR3/4–TRIF pathways, particularly when caspase-8 activity is insufficient. **(D)** Ferroptosis results from an imbalance between iron- and ACSL4-dependent phospholipid peroxidation and GPX4-mediated antioxidant defence, culminating in membrane-lipid damage and ferroptotic cell death. **(E)** Autophagy-associated cell-fate regulation depends on autophagic flux and cargo selectivity: PINK1–Parkin-mediated mitophagy generally removes damaged mitochondria and limits oxidative stress, whereas NCOA4-mediated ferritinophagy increases the labile iron pool and ferroptotic susceptibility. The relative importance of these pathways varies among osteoarthritis, intervertebral disc degeneration and osteoporosis according to cell lineage, anatomical niche and disease context. Dashed arrows indicate context-dependent regulatory relationships rather than obligatory conversion or direct molecular crosstalk between death programmes. Abbreviations: ACSL4, acyl-CoA synthetase long-chain family member 4; APAF1, apoptotic protease-activating factor 1; BAK, BCL-2 antagonist/killer; BAX, BCL-2-associated X protein; FADD, Fas-associated protein with death domain; GPX4, glutathione peroxidase 4; GSDMD, gasdermin D; IL, interleukin; IVDD, intervertebral disc degeneration; MLKL, mixed-lineage kinase domain-like protein; MOMP, mitochondrial outer-membrane permeabilization; NCOA4, nuclear receptor coactivator 4; NLRP3, NLR family pyrin domain containing 3; OA, osteoarthritis; OP, osteoporosis; PINK1, PTEN-induced kinase 1; PUFA, polyunsaturated fatty acid; RIPK, receptor-interacting protein kinase; ROS, reactive oxygen species; TLR, Toll-like receptor; TNFR1, tumour-necrosis-factor receptor 1; TRIF, TIR-domain-containing adaptor-inducing interferon-β.

Degenerative skeletal tissues are exposed to overlapping inflammatory, oxidative, mitochondrial and metabolic stresses that modify the activation thresholds of multiple regulated cell-death pathways. As summarized in Figure 1, apoptosis, pyroptosis, necroptosis and ferroptosis are mediated by distinct execution mechanisms, whereas autophagy-associated processes regulate cellular susceptibility in a flux-, cargo- and context-dependent manner. These pathways may be activated in parallel or regulated by shared upstream stress signals; however, the simultaneous detection of multiple pathway-associated markers does not necessarily indicate direct molecular crosstalk or conversion between death programmes.

### 2.1 Apoptosis

Apoptosis is a regulated, predominantly non-lytic form of cell death characterized by cell shrinkage, chromatin condensation, nuclear fragmentation and formation of apoptotic bodies. It is mediated mainly through extrinsic death-receptor and intrinsic mitochondrial pathways that converge on caspase activation (Vitale et al., 2023). In degenerative skeletal diseases, apoptosis contributes to the depletion of matrix-producing or bone-forming cells, but its pathological significance depends on the affected cell type, anatomical compartment and disease stage. In OA, apoptosis has been investigated primarily in articular chondrocytes. Taxifolin activates the Nrf2–HO-1 axis and reduces oxidative stress, apoptosis and extracellular matrix degradation (Jiang et al., 2023). Trachelogenin promotes HIF-1α-dependent glycolysis and chondrocyte survival (Jiang T. et al., 2024), whereas neurotrophin-3(NT3)–tropomyosin receptor kinase C (TrkC) signalling supports cartilage homeostasis through PI3K–Akt activation (Chang et al., 2025). These findings link chondrocyte apoptosis to oxidative stress, metabolic adaptation and impaired matrix maintenance. However, most evidence is derived from cellular and animal models, and the temporal contribution of apoptosis to early human OA remains incompletely defined.

In IVDD, apoptosis is most consistently documented in nucleus pulposus cells. Grem1 silencing reduces apoptosis and matrix disruption through restoration of TGF-β–Smad2/3 signalling (Chen et al., 2022). Procyanidin B2 activates PI3K–Akt–Nrf2 signalling (Zou et al., 2023), vitamin D suppresses TNF-α–NF-κB-associated apoptosis in human nucleus pulposus cells (Zhang et al., 2021), and tetrahedral-framework-nucleic-acid delivery of miR-155 modulates the BCL2–Bcl-2-associated X protein (BAX) axis and attenuates experimental disc degeneration (LI Z. et al., 2024). These studies support apoptosis as a common execution outcome of inflammatory signalling, oxidative injury and mitochondrial dysfunction rather than as a uniformly established initiating event. In OP, the consequence of apoptosis is strongly lineage-dependent. Apoptosis of osteoblasts, osteocytes or bone marrow mesenchymal stem cells (BMSCs) generally impairs bone formation and skeletal maintenance. By contrast, apoptosis of mature osteoclasts may restrict bone resorption. Apoptosis should therefore not be interpreted as uniformly detrimental across all bone-cell populations. Apoptosis represents a recurrent mechanism linking microenvironmental stress to cellular depletion, but its position as a driver, amplifier or downstream consequence differs among OA, IVDD and OP.

### 2.2 Pyroptosis

Pyroptosis is a gasdermin-dependent lytic form of regulated cell death characterized by membrane-pore formation, cell swelling and loss of plasma-membrane integrity. Canonical inflammasome activation promotes caspase-1-dependent processing of gasdermin D (GSDMD), IL-1β and IL-18, although cytokine maturation and gasdermin activation are not equivalent in every experimental context (Broz, 2025). Evidence of pyroptosis should therefore include gasdermin cleavage or pore formation in addition to inflammasome-associated markers. In OA, pyroptosis-associated signalling has been linked to chondrocyte injury and matrix degradation. α-Solanine suppresses NF-κB signalling, pyroptosis-associated markers and structural degeneration in experimental OA (Zhou et al., 2024). Interferon regulatory factor 1(IRF1)-dependent upregulation of guanylate-binding protein 5 (GBP5) enhances NLRP3 activation and chondrocyte injury (Tang et al., 2024), whereas the enhancer of zeste homolog 2 (EZH2)–miR-142-3p–high mobility group box 1(HMGB1) axis links epigenetic regulation and ER stress to inflammasome-associated cartilage degeneration (Chen Y. et al., 2025). Tetrahedral framework nucleic acids also reduce oxidative stress, pyroptosis-associated changes and matrix catabolism (Li J. et al., 2024). These findings support pyroptosis as an inflammatory amplifier, although studies targeting common upstream pathways do not necessarily establish that pyroptosis is the primary therapeutic mechanism.

In IVDD, evidence is concentrated in nucleus pulposus cells. MFG-E8 suppresses Nrf2–thioredoxin-interacting protein (TXNIP)–NLRP3-associated pyroptosis and extracellular matrix degradation (Ma et al., 2022). BMP7 reduces inflammasome activation in diabetes-associated disc degeneration (Yu et al., 2023), and acid-sensing ion channels promote NLRP3-associated inflammatory injury under acidic conditions (Zhao et al., 2021). When gasdermin activation or pathway-specific rescue is not demonstrated, these findings should be interpreted as inflammasome- or pyroptosis-associated signalling rather than definitive pyroptotic execution. In OP, pyroptosis-associated effects vary among osteocytes, osteoblasts and osteoclast-related signalling networks. Urolithin A suppresses receptor activator of nuclear factor kappa-B ligand (RANKL)-induced osteoclastogenesis and inflammatory signalling (Tao et al., 2021), although the specific requirement for pyroptotic execution requires further validation. Irisin reduces oxidative stress-associated osteocyte pyroptosis in diabetic OP models (Behera et al., 2022), whereas osteoclast-derived exosomal miR-30a-3p induces pyroptosis-associated injury in osteoblasts and aggravates lead-induced bone loss (Gao et al., 2025). These findings suggest that pyroptotic signalling may influence bone-cell communication and remodelling, but current evidence remains predominantly preclinical. Collectively, pyroptosis is best interpreted as a context-dependent inflammatory amplifier and tissue-injury effector rather than a universally established initiator of degenerative skeletal disease. Claims that it acts as a primary driver require temporal intervention, pathway-specific perturbation and functional rescue.

### 2.3 Ferroptosis

Ferroptosis is an iron-dependent form of regulated cell death driven by phospholipid peroxidation and failure of cellular antioxidant defence (Zheng and Conrad, 2025). Its identification requires integrated evidence of lipid peroxidation, iron dependency, disruption of ferroptosis-regulatory systems and rescue by ferroptosis-directed interventions rather than changes in glutathione peroxidase 4 (GPX4) or solute carrier family 7 member 11 (SLC7A11) expression alone. In OA, ferroptosis has been studied predominantly in articular chondrocytes. Human OA chondrocytes exposed to IL-1β show iron accumulation, lipid peroxidation and reduced GPX4 and SLC7A11 expression, with partial rescue by ferrostatin-1 (Xu W. et al., 2023). NADPH oxidase (NOX)1-mediated oxidative stress increases ferroptotic susceptibility through suppression of Nrf2–HO-1 signalling (Tao et al., 2024). Paeonol reduces acyl-CoA synthetase long-chain family member 4 (ACSL4)-associated ferroptosis, inflammatory responses and extracellular matrix degradation in cellular and animal models (Cao et al., 2025). These studies support a functional contribution of ferroptosis to chondrocyte injury, although its role in disease initiation remains uncertain.

In IVDD, ferroptotic susceptibility is associated with disturbed iron handling, oxidative stress and mitochondrial dysfunction. Ferroportin-mediated iron export protects nucleus pulposus cells and attenuates experimental disc degeneration (Lu et al., 2021). Iron overload induces ferroptosis-associated injury in cartilage endplate chondrocytes, which is reduced by deferoxamine, N-acetylcysteine and ferrostatin-1 (Wang W. et al., 2022). Humanin preserves mitochondrial homeostasis and reduces ferroptotic injury through HSP27-, JAK2–STAT3-and NF-κB-associated regulation (Zhu et al., 2025a). Evidence from annulus fibrosus cells remains comparatively limited. In OP, ferroptosis has distinct effects according to bone-cell lineage. Ferroptosis of osteoblasts and osteocytes generally impairs osteogenic function, osteocyte viability and skeletal integrity in diabetic, oestrogen-deficiency and iron-overload models (Jiang Z. et al., 2024; Yang Y. et al., 2022; J et al., 2022). Conversely, suppression of osteoclast ferroptosis through IRF9–STAT3 signalling promotes osteoclast differentiation (Lan et al., 2024). Thus, inhibition of ferroptosis may protect bone-forming cells while potentially prolonging the survival of bone-resorbing cells. Ferroptosis should therefore be interpreted as a cell- and disease-context-dependent regulator rather than a universally deleterious pathway. Longitudinal human evidence is still required to establish whether ferroptosis initiates disease or predominantly amplifies established tissue degeneration.

### 2.4 Autophagy-associated cell-fate regulation and ER stress

Autophagy is a lysosome-dependent homeostatic process that removes damaged proteins and organelles and supports adaptation to metabolic, oxidative and proteotoxic stress. Changes in microtubule-associated protein 1 light chain 3 (LC3), p62 or autophagosome abundance alone are insufficient to establish autophagy-dependent death. ER stress activates the unfolded protein response when ER protein-folding capacity is exceeded. Transient activation may restore proteostasis, whereas sustained ER stress can promote inflammation, mitochondrial dysfunction and cell death (Zhang SX. et al., 2024; Lin et al., 2007). In OA, most evidence supports autophagy as an adaptive quality-control mechanism. Ajugol activates TFEB-dependent autophagy and reduces ER stress, apoptosis and matrix degradation (Wu et al., 2023). GCN2 enhances autophagic flux and restrains inflammasome activation and cartilage injury (Fang et al., 2024), whereas koumine promotes phosphatase and tensin homolog (PTEN)-induced putative kinase 1(PINK1)–Parkin-dependent mitophagy and preserves mitochondrial function (Kong et al., 2024). These findings support a protective role for intact autophagic flux, although this role may vary with disease stage and selective cargo.

In IVDD, autophagy occurs within a hypoxic, acidic and nutrient-limited environment. Eicosapentaenoic acid promotes autophagy, suppresses ER stress and reduces nucleus pulposus-cell apoptosis (Lin et al., 2021). Under hypoxia and serum deprivation, lipopolysaccharide induces concurrent ER-stress-, autophagy- and apoptosis-associated changes, but this coexistence does not prove autophagy-dependent death (Dai et al., 2024). Palmatine activates TFEB-mediated autophagy and reduces ER stress, apoptosis and matrix degradation (Yu H. et al., 2025). Current evidence therefore supports autophagy predominantly as a compensatory response in nucleus pulposus cells. In OP, the effects of autophagy and ER stress depend on bone-cell identity. Resveratrol activates AMP-activated protein kinase (AMPK)–c-Jun N-terminal kinase 1 (JNK1)-associated autophagy and reduces osteocyte apoptosis in ovariectomized models (Wei et al., 2023). Inhibition of ER stress with 4-phenylbutyric acid improves bone microarchitecture in disuse OP (Al-Daghestani et al., 2024). PMAIP1 suppresses AMPK–mechanistic target of rapamycin (mTOR)-regulated autophagy and impairs osteoblast proliferation and differentiation (GAO Y. et al., 2024). Nevertheless, findings from osteoblasts, osteocytes and osteoclasts should not be treated as biologically interchangeable.

Autophagy functions primarily as an adaptive and cell-fate-regulating process. Mitophagy commonly limits mitochondrial and oxidative injury, whereas NCOA4-dependent ferritinophagy can increase labile iron and promote ferroptosis. The outcome therefore depends on autophagic flux, selective cargo, cell identity, anatomical niche and disease stage. Therapeutic modulation should focus on restoring appropriate flux or targeting specific selective-autophagy pathways rather than indiscriminately activating or inhibiting autophagy.

### 2.5 Disease-specific hierarchy of PCD roles

The functional position of a cell-death programme differs among OA, IVDD and OP. In OA, the strongest mechanistic evidence is concentrated in articular chondrocytes. Apoptosis contributes to depletion of matrix-maintaining cells (Chang et al., 2025; Yin et al., 2023). Pyroptosis amplifies inflammatory signalling and matrix catabolism through IRF1–GBP5–NLRP3-associated mechanisms (Tang et al., 2024), whereas receptor-interacting serine/threonine-protein kinase 1 (RIPK1)–receptor-interacting serine/threonine-protein kinase 3 (RIPK3)-associated necroptotic signalling may contribute to inflammatory and structural injury in experimental OA (Sun et al., 2023b; Piao et al., 2023). Ferroptosis has comparatively strong functional support from inhibitor-rescue experiments and regulation of the JNK–JUN–NCOA4 axis (Sun et al., 2023a). Macroautophagy and mitophagy are generally adaptive, although ferritinophagy may promote ferroptosis (Sun et al., 2023a; Fang et al., 2024; Kuwahara et al., 2022). In IVDD, nucleus pulposus-cell apoptosis is commonly an execution outcome of inflammatory, oxidative, mitochondrial and ER stress (Chen et al., 2022; Zou et al., 2023; Zhang et al., 2021; Li Z. et al., 2024). Pyroptosis reinforces inflammatory matrix degradation through Nrf2–TXNIP–NLRP3- and acid-sensing-ion-channel-associated mechanisms (Ma et al., 2022; Zhao et al., 2021), whereas necroptosis is associated with RIPK1–RIPK3–MLKL signalling, mitochondrial dysfunction and oxidative stress in nucleus pulposus cells (Cao et al., 2022; Fan et al., 2022; Zhu et al., 2026). Ferroptosis has been demonstrated in nucleus pulposus cells and cartilaginous endplate chondrocytes (Lu et al., 2021; Wang W. et al., 2022). Mitophagy generally provides compensatory mitochondrial quality control through VMP1–PINK1/Parkin-, HIF-1α-, DJ-1–HK2- and PINK1-associated mechanisms (Lin J. et al., 2024; Wang et al., 2018; Zhang Y. et al., 2025; Su C. et al., 2025). Evidence from annulus fibrosus cells remains substantially less extensive. In OP, cell lineage determines the tissue-level consequence of death. Apoptosis of osteoblasts and osteocytes (Wei et al., 2023), as well as ferroptosis of osteoblast-lineage cells and osteocytes (Jiang Z. et al., 2024; Zhang et al., 2023; Wang X. et al., 2022), generally impairs bone formation and skeletal integrity. Osteocyte-targeted GPX4 deletion provides relatively strong causal evidence linking enhanced osteocytic ferroptosis to RANKL upregulation, osteoclastogenesis and bone loss (Jiang Z. et al., 2024). Conversely, apoptosis of mature osteoclasts may restrict bone resorption (Kameda et al., 1997), whereas suppression of osteoclast ferroptosis may promote osteoclast differentiation (Ni et al., 2021).

The strength and cellular distribution of evidence differ substantially across diseases. In OA, the most consistent mechanistic evidence concerns apoptosis, pyroptosis, necroptosis and ferroptosis in articular chondrocytes, whereas synovial and subchondral-bone compartments are less comprehensively characterized. In IVDD, most mechanistic studies focus on nucleus pulposus (NP) cells, with more limited evidence from annulus fibrosus and cartilage endplate cells. In OP, evidence is distributed among osteoblasts, osteocytes, osteoclasts and BMSCs, and the same death pathway may exert opposing tissue-level effects according to the affected lineage.

## 3 Molecular crosstalk among PCD pathways

### 3.1 Apoptosis–pyroptosis relationships and multimodal PCD activation

In OA, apoptosis- and pyroptosis-associated changes are frequently detected in parallel, but their coexistence or simultaneous suppression does not establish direct molecular crosstalk. Morroniside reduces apoptotic and pyroptotic readouts while inhibiting NF-κB signalling, supporting regulation by a shared inflammatory input (YU et al., 2021). Monotropein similarly decreases apoptosis-, pyroptosis- and extracellular-matrix-degradation-associated markers; because causal dependence between the two execution pathways was not examined, these findings are more appropriately classified as functional coexistence (Li Z. et al., 2023). Isoliensinine attenuates oxidative stress and mitogen-activated protein kinase (MAPK)–NF-κB signalling together with both death-associated phenotypes, providing further evidence of convergent upstream regulation (Zhang Z. et al., 2024). Available OA studies therefore predominantly support parallel regulation by inflammatory and oxidative-stress pathways rather than direct apoptosis–pyroptosis interdependence. In IVDD, apoptosis-, pyroptosis- and necroptosis-associated responses may occur within the same pathological setting, although the strength of mechanistic evidence varies. MiR-155 reduces apoptotic and pyroptotic markers and extracellular matrix degradation in cholesterol-overloaded nucleus pulposus cells by targeting RORα, suggesting that metabolic stress regulates several injury pathways through a shared upstream mechanism (Qin et al., 2023). Inhibition of the ERK1/2–Drp1 axis similarly suppresses apoptosis- and pyroptosis-associated responses, supporting mitochondrial dysfunction as a shared regulatory node (SU S. et al., 2025). Kongensin A reduces multiple death-associated responses through transforming growth factor-β-activated kinase 1 (TAK1) inhibition, whereas RIPK1 inhibition suppresses apoptosis-, pyroptosis- and necroptosis-associated markers and attenuates experimental IVDD (Chen Y. et al., 2024; Zhu Z. et al., 2025). These findings identify TAK1 and RIPK1 as higher-order regulatory nodes. However, inhibition of a common upstream regulator does not establish PANoptosome-dependent PANoptosis. In the absence of coordinated execution-branch dependency, pathway-specific rescue or PANoptosome-related complex formation, these findings should be described as coordinated multimodal cell-death-associated activation.

In inflammatory osteoblast models relevant to impaired bone formation, TNF-α activates the ELP2–NLRP3–GSDMD/GSDME axis and induces lytic cell injury during osteogenic differentiation (Xia C. et al., 2023). GSDME involvement provides a potential executor-level connection between caspase activation and pyroptotic membrane permeabilization. Nevertheless, apoptosis-to-pyroptosis conversion should be concluded only when caspase-3-dependent GSDME cleavage is necessary for the lytic phenotype and genetic or pharmacological disruption of this link prevents the conversion. MiR-18a–HIF-1α signalling has been associated with concurrent apoptotic, pyroptotic and necroptotic changes during TNF-α-mediated impairment of osteogenic differentiation (Xia CL. et al., 2025). A separate ceRNA-network study identified lncRNA MIR17HG as a PANoptosis-related regulator and implicated it primarily in TNF-α-induced osteoblast apoptosis and inflammatory signalling (Li JX. et al., 2024). These observations support inflammatory activation of multiple death-associated pathways but do not constitute definitive evidence of PANoptosis without integrated dependency among the execution branches. The evidence for apoptosis–pyroptosis relationships in degenerative skeletal diseases is mechanistically heterogeneous. Most current studies support functional coexistence or regulation by shared inflammatory, metabolic and mitochondrial inputs, whereas direct executor-level conversion and integrated cell-death execution have been demonstrated less extensively.

### 3.2 Selective-autophagy-dependent regulation of ferroptosis

Selective autophagy regulates ferroptotic susceptibility through cargo- and cell-specific mechanisms. NCOA4-dependent ferritinophagy directly increases the labile iron pool by delivering ferritin to lysosomes (Sun et al., 2023a), whereas mitophagy generally limits mitochondrial reactive oxygen species and indirectly reduces lipid-peroxidation stress (Zhang Y. et al., 2025; SU C. et al., 2025; Wang H. et al., 2026). Chaperone-mediated autophagy may influence ferroptotic susceptibility through lysosomal degradation of specific proteins involved in redox regulation or iron metabolism (Wu et al., 2025). These mechanisms should not be treated as equivalent forms of autophagy–ferroptosis interaction. In OA, the strongest evidence of direct coupling involves ferritinophagy. Activation of the JNK–JUN–NCOA4 axis promotes ferritin degradation, increases intracellular iron availability and enhances chondrocyte ferroptosis, thereby aggravating extracellular matrix loss and joint degeneration (Sun et al., 2023a). By contrast, MK8722 activates SESN2–BNIP3-associated mitophagy and reduces ferroptotic injury, supporting an indirect protective effect of mitochondrial quality control (Wang H. et al., 2025). Urolithin B suppresses NCOA4-dependent ferritinophagy through the FGFR3–NCOA4–ferritin heavy chain 1(FTH1) axis, preserves iron storage and reduces chondrocyte ferroptosis (Li et al., 2025a). Thus, the effect of selective autophagy depends on its cargo: ferritinophagy can promote ferroptosis, whereas mitophagy generally limits oxidative and ferroptotic stress.

In IVDD, mitochondrial and chaperone-mediated quality-control pathways appear predominantly protective. UCHL1-mediated deubiquitination of HSPA8 enhances chaperone-mediated autophagy and reduces ferroptosis-associated nucleus pulposus-cell senescence and disc degeneration (Wu et al., 2025). VMP1 promotes PINK1–Parkin-dependent mitophagy, preserves mitochondrial function and reduces ferroptotic susceptibility (Zhang Y. et al., 2025). Ferristatin II has also been reported to suppress ferroptosis while enhancing HIF-1α-associated mitophagy in experimental IVDD (Su C. et al., 2025). These studies suggest that ferroptotic susceptibility is influenced by mitochondrial quality-control failure, although direct temporal dependency between mitophagy impairment and ferroptotic execution remains to be established in human disc tissues. In OP, selective-autophagy effects are strongly lineage-dependent. Hypoxia inhibits RANKL-induced ferritinophagy and protects osteoclasts from ferroptosis, suggesting that ferritinophagy influences osteoclast survival and potentially bone resorption (Ni et al., 2021). Endothelial-cell-derived exosomes reduce glucocorticoid-induced bone loss while suppressing ferritinophagy-associated osteoblast ferroptosis (Yang et al., 2021). Depletion of mitochondrial ferritin promotes mitochondrial dysfunction, mitophagy-associated changes and ferroptotic injury in osteoblasts, thereby impairing bone formation in diabetic OP (Wang X. et al., 2022). Restoration of SIRT3 activity has also been associated with reduced excessive mitophagy and ferroptotic injury in glucocorticoid-induced OP (Hu et al., 2025). These findings indicate that both deficient mitochondrial quality control and excessive mitochondrial removal may be harmful, depending on the cellular and metabolic context.

The strongest evidence for direct autophagy–ferroptosis coupling involves NCOA4-dependent ferritinophagy, in which ferritin degradation increases iron availability and ferroptotic susceptibility. By contrast, the relationship between mitophagy and ferroptosis is generally antagonistic, because mitochondrial quality control limits oxidative injury, although excessive or dysregulated mitophagy may also compromise cellular homeostasis. Ferroptosis and autophagy should therefore not be treated as two uniformly coordinated death programmes. Instead, selective autophagy acts as a context-dependent regulator of ferroptotic susceptibility, with effects determined by cargo selectivity, autophagic flux, cell identity and disease stage.

### 3.3 Necroptosis and inflammatory amplification

Necroptosis is a regulated lytic form of cell death executed by RIPK3-dependent phosphorylation and oligomerization of mixed-lineage kinase domain-like protein (MLKL). RIPK1 commonly participates in upstream signalling, although necroptosis can occur through RIPK1-independent mechanisms. Following phosphorylated MLKL membrane translocation, loss of plasma-membrane integrity permits the release of intracellular contents and damage-associated molecular patterns (Nakano, 2024). Evidence of necroptotic execution should ideally include MLKL phosphorylation or membrane translocation together with pathway-specific genetic or pharmacological rescue. In OA, preclinical evidence links RIPK1–RIPK3–MLKL-associated signalling to cartilage and synovial injury. TRADD promotes TNF-α-induced chondrocyte necroptosis through RIPK1–TAK1-associated signalling, whereas TRADD inhibition reduces necroptotic markers, inflammatory responses and cartilage degeneration (Sun et al., 2023b). DCC-2036 inhibits RIPK1 and RIPK3 kinase activity and attenuates necroptosis-associated changes in chondrocytes and synovial cells in experimental OA (Piao et al., 2023). These findings support necroptosis as a potential inflammatory and structural-damage amplifier but do not establish it as a universal initiating event.

In IVDD, RIPK1–RIPK3–MLKL-associated activation has been demonstrated predominantly in nucleus pulposus cells exposed to inflammatory stress. Pathway inhibition reduces cell death, mitochondrial dysfunction and oxidative injury (Cao et al., 2022). MyD88-associated signalling regulates ATP depletion, reactive oxygen species accumulation and necroptosis-associated injury, suggesting that inflammatory and mitochondrial signals converge upstream of execution (Fan et al., 2022). PHB2-dependent mitophagy preserves mitochondrial integrity and suppresses necroptosis-associated responses through PINK1–Parkin-dependent quality control (Zhu et al., 2026). Evidence remains primarily preclinical and is considerably more extensive in nucleus pulposus than in annulus fibrosus or cartilage endplate cells. In OP, necroptosis-associated effects vary according to bone-cell identity. RIPK3-associated necroptotic signalling has been linked to osteocyte death and bone loss after ovariectomy (He et al., 2021). Chronic ethanol exposure activates RIPK1–RIPK3–MLKL-associated signalling in osteoblasts, impairs osteogenic differentiation and contributes to osteopenia in experimental models (Guo et al., 2021). However, the specific contribution of necroptotic execution, as distinct from other forms of lytic osteocyte or osteoblast death, remains insufficiently resolved. Taken together, necroptosis may amplify skeletal degeneration by coupling RIPK1–RIPK3–MLKL-dependent cell loss to mitochondrial dysfunction, inflammatory signalling and damage-associated molecular-pattern release. However, RIPK1 and RIPK3 also possess non-necroptotic signalling functions, and pharmacological inhibition of these kinases does not by itself prove that therapeutic effects are mediated exclusively through blockade of necroptotic execution (Nakano, 2024).

### 3.4 Spatiotemporal and lineage-dependent control of multimodal cell death

**FIGURE 2:**
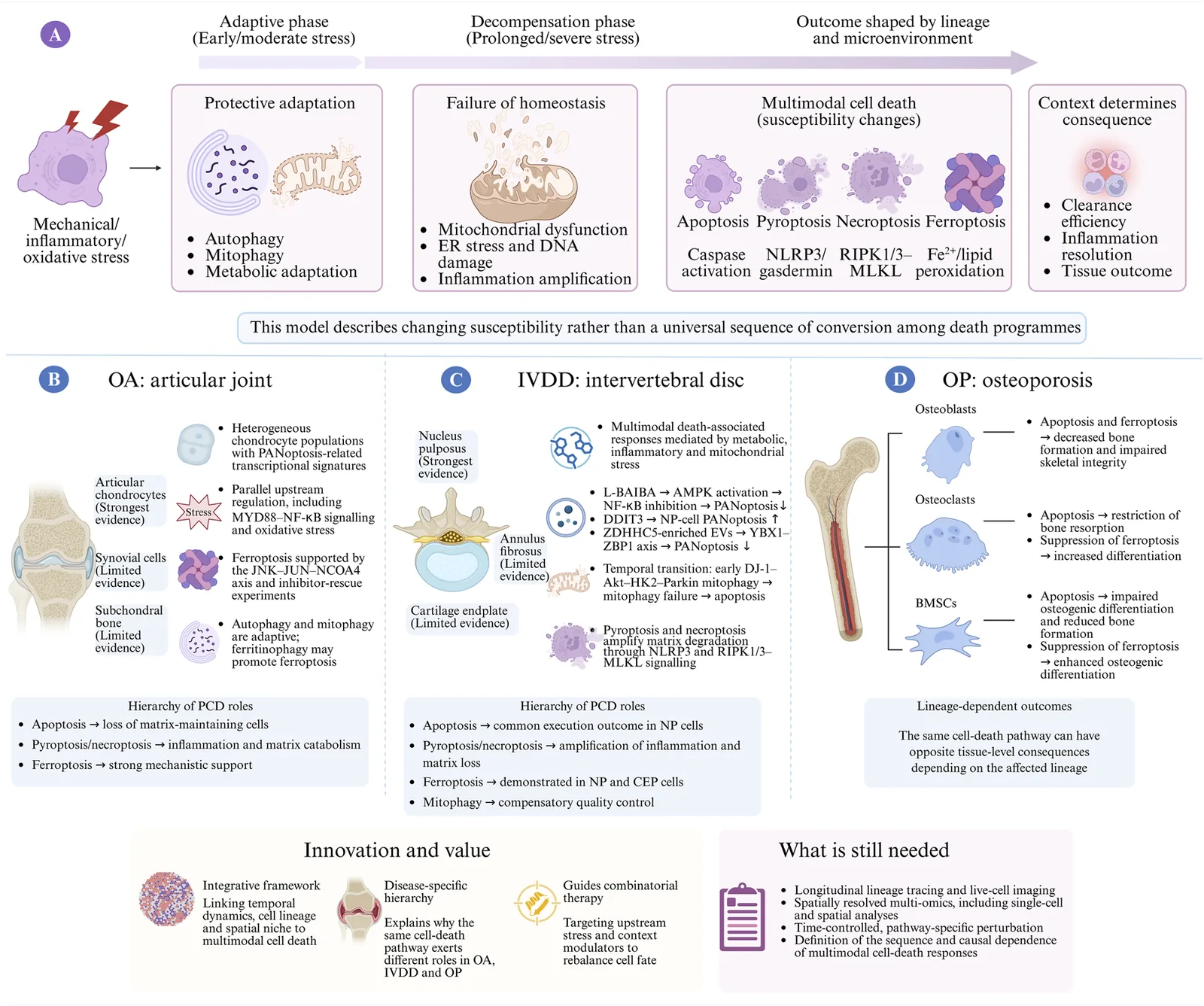
Spatiotemporal, lineage- and disease-context-dependent regulation of cell-death susceptibility in degenerative skeletal diseases. **(A)** Mechanical, inflammatory and oxidative stresses initially activate adaptive responses, including autophagy, mitophagy and metabolic reprogramming. With prolonged or severe stress, failure of cellular homeostasis leads to mitochondrial dysfunction, endoplasmic-reticulum stress, DNA damage and inflammatory amplification, thereby increasing susceptibility to apoptosis, pyroptosis, necroptosis and ferroptosis. The final tissue outcome is further determined by cell lineage, clearance efficiency, inflammatory resolution and the local microenvironment. This framework describes changing susceptibility rather than a universal sequence of conversion among cell-death programmes. **(B)** In osteoarthritis, the strongest mechanistic evidence is concentrated in articular chondrocytes, whereas evidence from synovial and subchondral-bone compartments remains less extensive. Heterogeneous chondrocyte populations exhibit distinct death-associated transcriptional states, and inflammatory or oxidative signals commonly regulate several pathways in parallel. Ferroptosis has relatively strong functional support from the JNK–JUN–NCOA4 axis and inhibitor-rescue experiments, whereas autophagy and mitophagy are generally adaptive and ferritinophagy may increase ferroptotic susceptibility. **(C)** In intervertebral disc degeneration, evidence is strongest in nucleus pulposus cells and more limited in annulus fibrosus and cartilage-endplate cells. Metabolic, inflammatory and mitochondrial stresses regulate multimodal death-associated responses through shared nodes, including AMPK–NF-κB, DDIT3 and ZBP1-associated signalling. Early DJ-1–AKT–HK2–Parkin-dependent mitophagy supports mitochondrial adaptation, whereas prolonged stress and failure of mitochondrial quality control increase apoptotic susceptibility. Pyroptosis- and necroptosis-associated signalling further amplify inflammation and extracellular-matrix degradation. **(D)** In osteoporosis, the tissue-level consequence of a cell-death pathway depends on the affected lineage. Apoptosis and ferroptosis in osteoblasts or bone marrow mesenchymal stem cells generally impair osteogenic differentiation and bone formation, whereas apoptosis of mature osteoclasts may restrict bone resorption. Conversely, suppression of osteoclast ferroptosis may enhance osteoclast differentiation. These lineage-dependent effects explain why the same pathway may produce opposing skeletal outcomes. Collectively, longitudinal, lineage-specific and spatially resolved studies are required to distinguish adaptive responses, shared upstream regulation, sequential transitions and direct molecular interdependence. Abbreviations: AKT, protein kinase B; AMPK, AMP-activated protein kinase; BMSC, bone marrow mesenchymal stem cell; CEP, cartilage endplate; DDIT3, DNA damage-inducible transcript 3; ER, endoplasmic reticulum; EV, extracellular vesicle; HK2, hexokinase 2; IVDD, intervertebral disc degeneration; JNK, c-Jun N-terminal kinase; L-BAIBA, L-β-aminoisobutyric acid; MLKL, mixed-lineage kinase domain-like protein; MYD88, myeloid differentiation primary response 88; NCOA4, nuclear receptor coactivator 4; NF-κB, nuclear factor-κB; NLRP3, NLR family pyrin domain containing 3; NP, nucleus pulposus; OA, osteoarthritis; OP, osteoporosis; PCD, programmed cell death; RIPK, receptor-interacting protein kinase; ZBP1, Z-DNA-binding protein 1; ZDHHC5, zinc finger DHHC-type palmitoyltransferase 5.

Representative nucleus pulposus-cell studies suggest that early oxidative stress can activate adaptive mitophagy, whereas prolonged stress and failure of mitochondrial quality control increase susceptibility to mitochondrial dysfunction, apoptosis and senescence (Lin J. et al., 2024; Wang et al., 2018). Whether comparable temporal transitions occur across OA, IVDD and OP remains to be established. In OA, current evidence supports cellular heterogeneity and coordinated regulation by shared upstream pathways more strongly than direct temporal conversion. MiR-203a-3p reduces apoptosis- and pyroptosis-associated readouts, oxidative stress and matrix degradation through MYD88–NF-κB signalling (CHEN J. et al., 2023). This demonstrates parallel upstream regulation but not apoptosis-to-pyroptosis conversion. Single-cell analyses have identified heterogeneous chondrocyte populations enriched in PANoptosis-associated transcriptional signatures and predicted responsiveness to andrographolide (Zhou D. et al., 2025). These data support cell-population heterogeneity, but transcriptional signatures and predicted drug responses are not equivalent to active PANoptotic execution. Spatial heterogeneity should be concluded only when supported by spatially resolved or histological evidence. As summarized in Figure 2, the susceptibility and consequences of multimodal cell-death responses vary with stress duration, cell lineage and the local microenvironment. These changes reflect context-dependent regulation rather than a universal sequence of conversion among death programmes.

In IVDD, multimodal death-associated responses are shaped by metabolic, inflammatory and mitochondrial stress. L-BAIBA reduces apoptosis-, pyroptosis- and necroptosis-associated markers and extracellular matrix degradation through AMPK–NF-κB-related signalling (Qin et al., 2024). DDIT3 promotes coordinated activation of multiple death-associated pathways under inflammatory stress, whereas engineered extracellular-vesicle delivery of ZDHHC5 suppresses ZBP1-associated multimodal responses (Wang K. et al., 2026; Chen T. et al., 2025). These findings identify shared stress-responsive regulators but do not demonstrate PANoptosome-dependent execution. More direct temporal evidence is provided by mitochondrial quality-control studies. During early oxidative stress, DJ-1 translocates to mitochondria, promotes AKT phosphorylation and HK2 recruitment, and facilitates Parkin-dependent removal of dysfunctional mitochondria. Under prolonged stress, DJ-1 depletion impairs mitophagy, promotes cytochrome-c release and increases caspase-dependent apoptosis (Lin J. et al., 2024). PINK1 depletion similarly impairs stress-induced mitophagy and aggravates mitochondrial dysfunction and senescence in human nucleus pulposus cells (Wang et al., 2018). These observations support a temporal transition from adaptive mitochondrial quality control to irreversible dysfunction and apoptosis, but not universal interconversion among distinct death-execution programmes.

Cell-fate consequences in bone are also lineage-dependent. TNF-α-induced miR-18a–HIF-1α signalling in osteoblasts is associated with concurrent apoptosis-, pyroptosis- and necroptosis-related changes and impaired osteogenic differentiation (Zhang et al., 2024). This supports inflammatory multimodal cell injury but not definitive PANoptosis. In a postnatal bone-remodelling model, repeated apoptosis of mature osteocalcin-positive osteoblasts stimulated macrophage efferocytosis, increased osteoblast precursors and enhanced vertebral trabecular bone formation, whereas apoptosis of osterix-positive cells did not produce the same response (Batoon et al., 2024). Because this study examined postnatal bone development rather than OP, it provides contextual evidence of lineage dependence rather than direct evidence of a protective role for apoptosis in osteoporosis. The efficiency of apoptotic-cell clearance further modifies tissue outcomes. In aged bone, impaired macrophage efferocytosis permits accumulation of apoptotic osteoblasts, promotes inflammatory signalling and contributes to bone loss (Xu R. et al., 2023). The same death programme may therefore have different consequences according to cell lineage, temporal pattern and the capacity of the local microenvironment to clear dying cells and resolve inflammation.​ Current evidence supports stage-, lineage- and context-dependent regulation of cell fate but does not establish universal conversion among apoptosis, pyroptosis, necroptosis and ferroptosis. Longitudinal lineage tracing, live-cell imaging, spatially resolved profiling and time-controlled pathway-specific perturbation are required to define the sequence and causal dependency of multimodal cell-death responses. Representative mechanistic relationships among regulated cell-death programmes and associated stress responses are summarized in Table 1.

**TABLE 1:** Representative mechanistic relationships among regulated cell-death programmes and associated stress responses in degenerative skeletal diseases.

Disease/principal cell type	Processes examined	Key regulator(s)	Experimental context	Evidence-supported relationship	Mechanistic interpretation and principal limitation	Ref.
osteoarthritis (OA)/articular chondrocytes	Autophagy and apoptosis	sirtuin 3(SIRT3); phosphoinositide 3-kinase (PI3K)–protein kinase B (AKT)–mechanistic target of rapamycin (mTOR)	interleukin-1 beta (IL-1β)-induced inflammation and mitochondrial stress	Shared upstream regulation with an antagonistic functional association	SIRT3-associated autophagy activation was accompanied by reduced apoptosis. This supports upstream coupling but not direct interaction between autophagic and apoptotic execution mechanisms	(Xu et al., 2021)
OA/articular chondrocytes	endoplasmic reticulum (ER) stress, mitochondrial dysfunction and apoptosis	AMP-activated protein kinase (AMPK)–SIRT3; peroxisome proliferator-activated receptor-γ coactivator 1α(PGC-1α); mitochondrial transcription factor A (TFAM); nuclear factor erythroid 2-related factor 2(NRF2); ER-stress proteins	tert-butyl hydroperoxide (TBHP)-induced oxidative and ER stress	Sequential organelle-stress regulation of apoptotic susceptibility	AMPK–SIRT3 activation improved mitochondrial function and reduced apoptosis-associated injury. The evidence concerns stress regulation rather than crosstalk between distinct death programmes	(Qiao et al., 2025)
OA/articular chondrocytes	Mitophagy-associated changes, apoptosis and inflammasome-associated inflammation	arachidonate 5-lipoxygenase (ALOX5); nuclear factor-κB (NF-κB); NLR family pyrin domain containing 3(NLRP3)	IL-1β-induced inflammatory and mitochondrial stress	Shared upstream regulation and functional coexistence	ALOX5 modulation altered mitophagy-, apoptosis- and inflammasome-associated readouts. Gasdermin-dependent execution and direct pathway interdependence were not established	(Zhou et al., 2025b)
OA/articular chondrocytes	Excessive mechanical-strain-associated apoptosis	piezo-type mechanosensitive ion channel component 1(Piezo1)–calcium ion (Ca^2+^)–calcineurin–nuclear factor of activated T cells 1(NFAT1)	Excessive mechanical strain	Mechanotransduction-mediated regulation of apoptotic susceptibility	This represents an upstream biomechanical input to chondrocyte apoptosis rather than direct crosstalk between cell-death programmes	(Ren et al., 2023)
OA/articular chondrocytes	Autophagy and apoptosis	Leptin; lysyl oxidase-like 3 (LOXL3)-associated signalling	Elevated leptin exposure	Potential antagonistic functional relationship	LOXL3-associated changes were accompanied by reduced autophagy-related activity and increased apoptosis. Dynamic autophagic-flux measurements and pathway-specific rescue are required to establish dependency	(Wei, 2022)
OA/articular chondrocytes	Inflammatory signalling, apoptosis and matrix catabolism	NRF2–high-mobility group box 1(HMGB1)–NF-κB	IL-1β-induced oxidative and inflammatory stress	Shared upstream regulation	NRF2 activation reduced inflammatory, apoptotic and matrix-degradative responses. This does not demonstrate interaction between distinct death-execution pathways	(Li et al., 2023d)
osteoporosis (OP)/osteoblasts	Epigenetic regulation of ferroptosis	DNA methyltransferase 1 (DNMT1)/DNA methyltransferase 3A (DNMT3A)/DNA methyltransferase 3B (DNMT3B)–glutathione peroxidase 4 (GPX4)	ovariectomy (OVX)-induced OP, human OP specimens and ferroptotic osteoblast models	Direct epigenetic regulation of ferroptotic susceptibility	GPX4-promoter hypermethylation links aberrant DNA methylation to osteoblast ferroptosis. The strongest causal interpretation requires methylation-specific manipulation and ferroptosis-directed rescue	(Ruan et al., 2024)
Age-related OP/osteoblasts	Ferroptosis and cellular senescence	vitamin D receptor (VDR)–NRF2–GPX4	D-galactose-associated ageing and oxidative stress	Functional coexistence through shared redox regulation	Reduced ferroptosis- and senescence-associated phenotypes do not establish bidirectional mechanistic dependence between ferroptosis and senescence	(Xu et al., 2022)
Postmenopausal OP/osteoblasts	gasdermin E (GSDME)-associated pyroptosis and mitochondrial apoptosis-associated changes	PI3K–AKT; GSDME	OVX-induced OP and osteoblast injury	Shared upstream regulation with a possible executor-level linkage	The study supports reduced mitochondrial injury and GSDME-associated pyroptosis. Apoptosis-to-pyroptosis conversion requires direct demonstration of caspase-3-dependent GSDME cleavage and pathway-specific rescue	(Chai et al., 2024)
OA/chondrocytes and osteoclast-lineage cells	Pyroptosis-associated inflammation and osteoclastogenesis	NF-κB–NLRP3–caspase-1(CASP1); receptor activator of NF-κB ligand (RANKL)–nuclear factor of activated T cells 1(NFATC1)	IL-1β-stimulated chondrocytes and RANKL-stimulated osteoclast-lineage cells	Parallel regulation in two cell populations through shared inflammatory signalling	The intervention affected chondrocyte pyroptosis-associated injury and osteoclastogenesis separately. Direct chondrocyte–osteoclast communication was not demonstrated	(Zheng et al., 2023)
intervertebral disc degeneration (IVDD)/nucleus pulposus cells	Pyroptosis-associated injury and extracellular matrix (ECM) dysregulation	PI3K–AKT–NF-κB–NLRP3	IL-1β-induced inflammatory stress	Shared upstream regulation	Suppression of inflammasome and ECM-catabolic markers supports pathway regulation. Gasdermin-dependent lytic execution should be confirmed before definitive pyroptosis is concluded	(Gong et al., 2023)
IVDD/nucleus pulposus cells	Chaperone-mediated autophagy, senescence and apoptosis	lysosome-associated membrane protein 2A (LAMP2A)–dual-specificity tyrosine-phosphorylation-regulated kinase 1A (DYRK1A)–forkhead box C1(FOXC1)	IL-1β stimulation and reduced chaperone-mediated autophagy	Potential sequential cell-fate regulation	A senescence-to-apoptosis switch requires time-resolved evidence and pathway-specific rescue. Concurrent changes in senescence and apoptosis markers alone are insufficient	(Cheng et al., 2025)
IVDD/nucleus pulposus cells	Autophagy and ferroptosis	peroxisome proliferator-activated receptor-γ(PPARγ)–AXL receptor tyrosine kinase (AXL)–PI3K–AKT; GPX4	Oxidative, inflammatory and iron-related stress	Autophagy-associated regulation of ferroptotic susceptibility	The study supports autophagy-mediated suppression of ferroptosis, but no specific selective-autophagy cargo was established. General autophagy activation should not be assumed to suppress ferroptosis uniformly	(Sun et al., 2025b)
OP/osteoblasts	Autophagy-associated apoptosis	DNA damage-regulated autophagy modulator (DRAM); autophagy-related proteins	Oestrogen-deficiency-associated stress	Autophagy-associated facilitation of apoptosis	Increased autophagy- and apoptosis-associated markers do not establish autophagy-dependent cell death without genetic, pharmacological or flux-based dependency testing	(Tang et al., 2019)
OVX-induced OP/bone tissue	Autophagy- and apoptosis-associated responses	caspase-3(CASP3); B-cell lymphoma 2(BCL2); microtubule-associated protein 1 light chain 3 (LC3); beclin 1(BECN1)	Oestrogen deficiency and morin intervention	Functional coexistence under shared stress regulation	Simultaneous pharmacological suppression of autophagy- and apoptosis-associated markers may reflect pleiotropic drug effects and does not prove cooperative execution	(Jiang et al., 2024c)
OA/articular chondrocytes	Therapeutic suppression of apoptosis-, necroptosis- and pyroptosis-associated markers	BCL-2-associated X protein (BAX)/BCL2; receptor-interacting protein kinase 3 (RIPK3); NLRP3–CASP1–gasdermin D (GSDMD)	reactive oxygen species (ROS)-responsive nanomicelle–hydrogel intervention under oxidative and inflammatory stress	Coordinated multimodal marker suppression during therapeutic perturbation	Simultaneous reduction of three marker sets should not be labelled PANoptosis without execution-branch dependency, pathway-specific rescue or PANoptosome-complex evidence	(Li et al., 2026)
OA/chondrocyte subpopulations	Multimodal inflammatory cell-death transcriptional signatures	caspase-8(CASP8); CASP1; Toll-like receptor 3 (TLR3); interleukin-18(IL18)	Bioinformatics and single-cell transcriptomic analysis of OA chondrocyte populations	Signature-level association and predicted shared regulatory nodes	Transcriptomic enrichment and predicted drug responsiveness do not establish active PANoptotic execution or PANoptosome formation	(Zhou et al., 2025a)

Abbreviations: AKT, protein kinase B; ALOX5, arachidonate 5-lipoxygenase; AMPK, AMP-activated protein kinase; AXL, AXL, receptor tyrosine kinase; BAX, BCL-2-associated X protein; BCL2, B-cell lymphoma 2; BECN1, beclin 1; Ca^2+^, calcium ion; CASP1, caspase-1; CASP3, caspase-3; CASP8, caspase-8; DNMT1, DNA, methyltransferase 1; DNMT3A, DNA, methyltransferase 3A; DNMT3B, DNA, methyltransferase 3B; DRAM, DNA, damage-regulated autophagy modulator; DYRK1A, dual-specificity tyrosine-phosphorylation-regulated kinase 1A; ECM, extracellular matrix; ER, endoplasmic reticulum; FOXC1, forkhead box C1; GPX4, glutathione peroxidase 4; GSDMD, gasdermin D; GSDME, gasdermin E; HMGB1, high-mobility group box 1; IL-1β, interleukin-1, beta; IL-18, interleukin-18; IVDD, intervertebral disc degeneration; LAMP2A, lysosome-associated membrane protein 2A; LC3, microtubule-associated protein 1 light chain 3; LOXL3, lysyl oxidase-like 3; mTOR, mechanistic target of rapamycin; NFAT1, nuclear factor of activated T cells 1; NFATC1, nuclear factor of activated T cells 1; NF-κB, nuclear factor-κB; NLRP3, NLR, family pyrin domain containing 3; NRF2, nuclear factor erythroid 2-related factor 2; OA, osteoarthritis; OP, osteoporosis; OVX, ovariectomy; PGC-1α, peroxisome proliferator-activated receptor-γ coactivator 1α; PI3K, phosphoinositide 3-kinase; Piezo1, piezo-type mechanosensitive ion channel component 1; PPARγ, peroxisome proliferator-activated receptor-γ; RANKL, receptor activator of NF-κB, ligand; RIPK3, receptor-interacting protein kinase 3; ROS, reactive oxygen species; SIRT3, sirtuin 3; TBHP, tert-butyl hydroperoxide; TFAM, mitochondrial transcription factor A; TLR3, Toll-like receptor 3; VDR, vitamin D receptor.

## 4 Cell-death-dependent remodelling of disease-specific skeletal microenvironments

**FIGURE 3:**
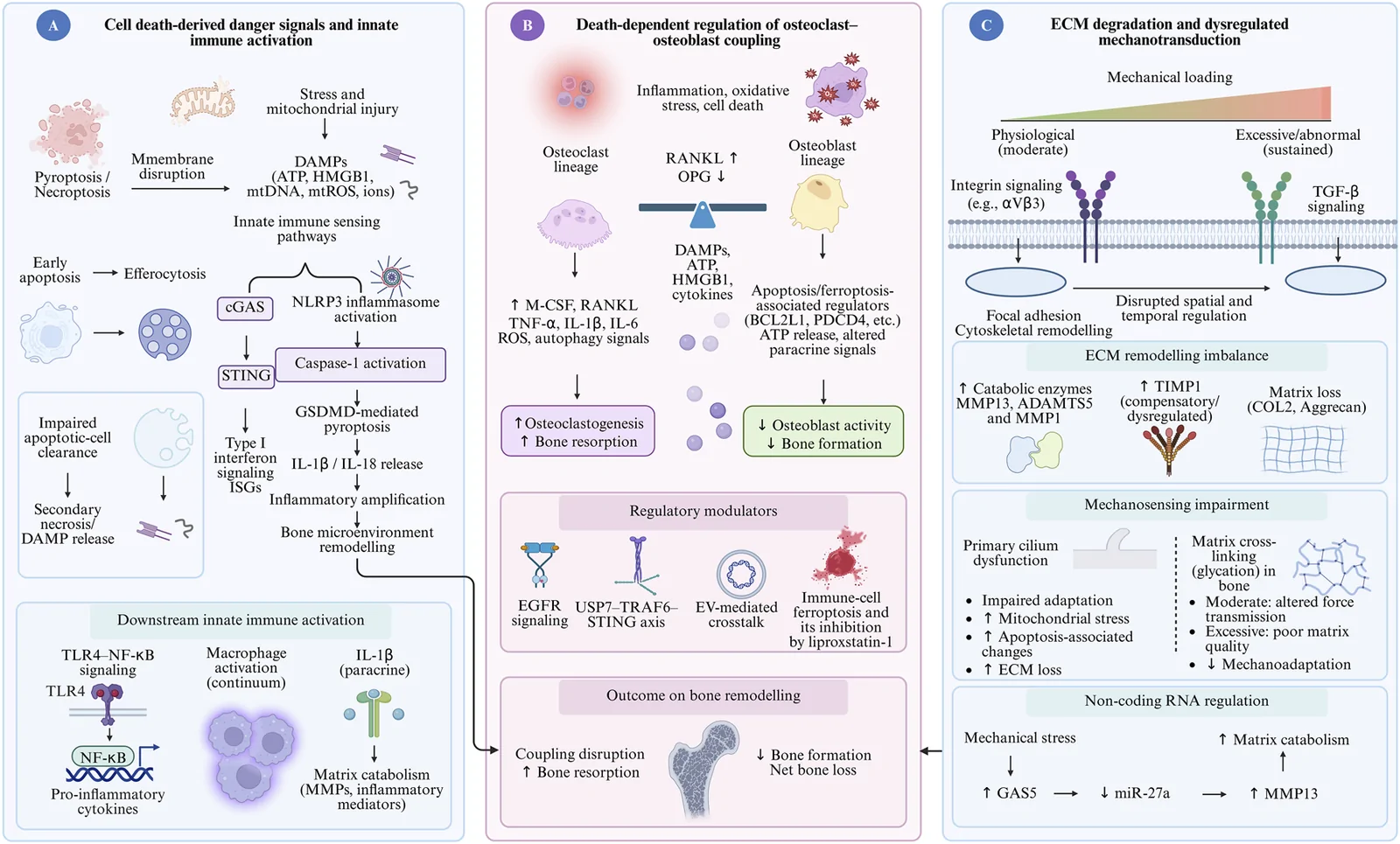
Programmed cell death-driven remodelling of disease-specific skeletal microenvironments. **(A)** Pyroptosis and necroptosis promote membrane disruption and the release of DAMPs, including extracellular ATP, HMGB1 and mitochondrial components. These DAMPs activate distinct innate immune-sensing pathways, including NLRP3-associated signalling and, in the case of cytosolic DNA, the cGAS–STING pathway. Early apoptotic cells are normally removed by efferocytosis, whereas impaired clearance can lead to secondary necrosis and additional DAMP release. These events amplify TLR4–NF-κB signalling, macrophage activation, cytokine production and matrix catabolism. **(B)** Inflammation, oxidative stress and lineage-specific cell death disrupt osteoclast–osteoblast coupling. Increased RANKL/OPG imbalance and osteoclastogenic signals enhance bone resorption, whereas apoptosis- or ferroptosis-associated injury to osteoblast-lineage cells reduces bone formation. EGFR signalling, the USP7–TRAF6–STING axis, extracellular-vesicle-mediated communication and immune-cell ferroptosis further modulate bone remodelling. **(C)** Physiological loading supports integrin-associated mechanotransduction and matrix homeostasis, whereas excessive or sustained loading disrupts integrin–TGF-β signalling, increases MMP1, MMP13 and ADAMTS5, alters TIMP1 responses and promotes loss of type II collagen and aggrecan. Primary-cilium dysfunction, glycation-associated matrix cross-linking and the GAS5–miR-27a–MMP13 axis further impair mechanosensing and enhance matrix degradation. Collectively, these processes form context-dependent feed-forward networks that promote skeletal degeneration. Abbreviations: ADAMTS5, a disintegrin and metalloproteinase with thrombospondin motifs 5; ATP, adenosine triphosphate; cGAS, cyclic GMP–AMP synthase; DAMPs, damage-associated molecular patterns; ECM, extracellular matrix; EGFR, epidermal growth factor receptor; EV, extracellular vesicle; GAS5, growth arrest-specific transcript 5; GSDMD, gasdermin D; HMGB1, high-mobility group box 1; MMP, matrix metalloproteinase; NF-κB, nuclear factor-κB; NLRP3, NLR family pyrin domain containing 3; OPG, osteoprotegerin; RANKL, receptor activator of NF-κB ligand; STING, stimulator of interferon genes; TGF-β, transforming growth factor-β; TIMP1, tissue inhibitor of metalloproteinases 1.

PCD contributes to degenerative skeletal diseases not only through the loss of tissue-maintaining cells but also by reshaping local inflammatory, remodelling and mechanical microenvironments. As summarized in Figure 3, lytic cell death and defective clearance of apoptotic cells amplify innate immune signalling; lineage-specific injury disrupts osteoclast–osteoblast coupling; and abnormal mechanical loading promotes extracellular-matrix catabolism and impaired mechanosensing. These processes form context-dependent feed-forward networks whose consequences vary according to cell identity, anatomical compartment and disease stage.

### 4.1 Cell-death-derived danger signals and innate immune activation

Regulated cell death can influence degenerative skeletal diseases not only through cellular depletion but also by modifying the local inflammatory environment. Lytic death programmes, particularly pyroptosis and necroptosis, compromise plasma-membrane integrity and permit the extracellular release of intracellular contents and DAMPs (Broz, 2025; Nakano, 2024; Kaczmarek et al., 2013). By contrast, apoptotic cells generally preserve membrane integrity during early execution, expose phagocytic signals and undergo efferocytic clearance (Vitale et al., 2023). When the rate of apoptosis exceeds local phagocytic capacity or apoptotic-cell clearance is impaired, apoptotic cells can progress to secondary necrosis, resulting in membrane rupture and the release of potentially inflammatory intracellular contents (Vivers et al., 2002). Immunogenic features may additionally modify the inflammatory consequences of apoptotic cell death. Different death programmes should therefore not be assumed to generate equivalent patterns of DAMP release.

Mitochondrial quality control may modify this process in a stress-duration-dependent manner. During relatively early oxidative stress, mitochondrial translocation of DJ-1 promotes AKT phosphorylation, HK2 recruitment and Parkin-dependent removal of dysfunctional mitochondria in nucleus pulposus cells. Under sustained stress or DJ-1 depletion, impaired mitophagy is associated with cytochrome-c release into the cytosol, caspase activation, apoptosis and extracellular matrix loss (Lin J. et al., 2024; Sun et al., 2021). This evidence supports a transition from adaptive mitochondrial quality control to apoptotic susceptibility, rather than demonstrating that mitochondrial apoptosis necessarily produces extensive extracellular DAMP release. Inflammatory mediators released or activated during cell injury can further alter neighbouring cells. In IL-1β-stimulated chondrocytes, increased matrix metalloproteinase (MMP)13 and a disintegrin and metalloproteinase with thrombospondin motifs 5 (ADAMTS5) expression and reduced type II collagen were observed, and these changes were attenuated by METRNL treatment (Liu J. et al., 2023). In experimental TMJOA, pharmacological inhibition of toll-like receptor 4 (TLR4) reduces MyD88–NF-κB–NLRP3 signalling, reactive oxygen species (ROS)-associated macrophage inflammation, chondrocyte pyroptosis, synovial inflammation and cartilage degeneration (Liu X. et al., 2023). Macrophage responses should nevertheless be regarded as a continuum of context-dependent activation states rather than a strict M1/M2 dichotomy. Mitochondrial injury, DAMP sensing, inflammasome activation and lytic cell death can form feed-forward inflammatory networks. The existence and relative importance of these networks differ among cartilage, intervertebral-disc and bone-remodelling compartments and should be evaluated using pathway-specific perturbation rather than marker co-expression alone.

### 4.2 Death-dependent regulation of osteoclast–osteoblast coupling

Osteoblast–osteoclast coupling is directly relevant to OP and to bone-remodelling compartments such as subchondral bone in OA, but it should not be generalized to all tissues affected by degenerative skeletal disease. Under physiological conditions, osteoclastic bone resorption and osteoblastic bone formation are coordinated through soluble factors, extracellular vesicles and contact-dependent signalling. Inflammation, oxidative stress and cell death can disrupt this coupling by altering the survival, differentiation and secretory profiles of bone-lineage cells. Osteoclast differentiation is driven primarily by macrophage colony-stimulating factor and RANKL and is further modified by inflammatory, redox and autophagy-related signals. TNF-α, IL-1β, IL-6 and excessive reactive oxygen species can enhance osteoclastogenic signalling in appropriate experimental contexts, whereas oestrogen deficiency increases RANKL-associated bone resorption. TET2-dependent regulation of autophagy has also been implicated in osteoclast differentiation and ovariectomy-associated bone loss (Yang C. et al., 2022). Ferroptosis-associated changes in immune cells may indirectly reshape the osteoimmune environment. In an experimental arthritis model, liproxstatin-1 preserved anti-inflammatory macrophage features, reduced HMGB1–TLR4–STAT3-associated signalling and attenuated joint injury (Feng Z. et al., 2024). Because liproxstatin-1 acts primarily as an inhibitor of lipid-peroxidation propagation, its effects should not be described simply as “protecting GPX4 function” unless GPX4 dependency was directly demonstrated. Moreover, the consequence of ferroptosis differs by lineage: ferroptotic injury to osteoblasts or osteocytes generally impairs bone formation, whereas ferroptosis in osteoclast-lineage cells may restrict their survival or differentiation.

Loss of osteoblast-lineage cells may disturb coupling through both reduced bone formation and altered paracrine signalling. In the cited model, reduced BCL2L1 expression promoted osteoblast apoptosis and extracellular ATP release, which enhanced osteoclastogenic activity (Moriishi et al., 2023). These findings suggest that osteoblast death can influence osteoclast behaviour, although such mechanisms should be interpreted within the specific models in which they were demonstrated. The RANKL–OPG balance is a major, but not exclusive, regulator of osteoblast–osteoclast coupling. In titanium-particle-induced osteolysis, inhibition of EGFR signalling suppresses pro-inflammatory macrophage polarization and RANKL-induced osteoclastogenesis through PI3K–AKT- and NF-κB-associated mechanisms (Jia et al., 2024). USP7 has been reported to suppress osteoclastogenesis through TRAF6 deubiquitination and stimulator of interferon genes (STING)-associated negative regulation of RANK–NF-κB–MAPK signalling (Xie Z. et al., 2023). Engineered EphA2-displaying exosomes have been used to deliver METTL14 to osteoclasts, increase NFATc1 m6A methylation and suppress osteoclast-mediated bone resorption (Yang J-G. et al., 2023). This study supports targeted exosomal delivery but does not by itself establish the extent of physiological extracellular-vesicle exchange between osteoblast- and osteoclast-lineage cells. Current evidence therefore supports a model in which cell death modifies bone remodelling through lineage loss, DAMP and ATP release, altered RANKL–OPG signalling and changes in intercellular communication. However, most supporting evidence is derived from cellular and animal models, and the temporal contribution of each pathway to human bone remodelling remains insufficiently defined.

### 4.3 Extracellular matrix (ECM) degradation and dysregulated mechanotransduction

Loss of balance between extracellular matrix synthesis and degradation is a defining feature of OA and IVDD. Mechanical loading contributes to cartilage and intervertebral-disc homeostasis, but its biological effect depends on loading magnitude, duration, frequency, tissue condition and cell type. Physiological loading can support matrix maintenance, whereas excessive, abnormal or sustained loading can promote inflammatory signalling, mitochondrial injury, cell-death susceptibility and extracellular matrix degradation. Integrin-dependent signalling links mechanical forces to intracellular responses, including TGF-β, focal-adhesion and cytoskeletal pathways. Because the effects of integrin αV and TGF-β signalling vary with disease stage and mechanical context, the relationship should not be described simply as activation or suppression. In a murine OA model, passive hydrotherapy preserved cartilage and muscle integrity and was associated with altered integrin αV–TGF-β mechanotransduction (Di et al., 2025). Whether this pathway broadly mediates the effects of abnormal mechanical loading requires further validation.

Matrix degradation is mediated principally by an imbalance between matrix-degrading enzymes and their endogenous inhibitors. MMP13 and ADAMTS5 contribute to collagen and aggrecan loss in cartilage and nucleus pulposus tissues. PMA stimulation increases ADAMTS5 and MMP1 expression in human nucleus pulposus cells and also alters TIMP1 expression (Quan and Kim, 2024). Because TIMP1 is an endogenous metalloproteinase inhibitor, its upregulation should be interpreted as part of a compensatory or dysregulated remodelling response rather than as a direct driver of matrix degradation. In degenerative nucleus pulposus cells, RNA interference targeting MMP3 and MMP13 increases type II collagen abundance, supporting a role for these enzymes in collagen-matrix loss (Mern and Thomé, 2024). In OA chondrocyte models, MOTS-c improves extracellular-matrix homeostasis and suppresses matrix-metalloproteinase production (Li K. et al., 2025). TB-II reduces IL-1β-induced MMP1, MMP3 and MMP13 expression through inhibition of MAPK and NF-κB signalling *in vitro* (Liu et al., 2022).

Non-coding RNAs can connect mechanical stress to extracellular matrix regulation. The GAS5–miR-27a–MMP13 network has been implicated in mechanically induced matrix catabolism: reduced miR-27a activity is associated with increased MMP13 expression, whereas restoration of miR-27a reduces catabolic signalling and supports COL2A1 expression (Zheng T. et al., 2021). These observations demonstrate regulation of matrix turnover but do not, by themselves, establish direct crosstalk between distinct cell-death execution pathways. Primary cilia are important mechanosensory organelles in chondrocytes, nucleus pulposus cells and osteocytes. Disruption of primary-cilium-dependent signalling can impair adaptive responses to mechanical stimulation and may increase mitochondrial dysfunction, apoptosis-associated changes and matrix loss. These effects are context-dependent and should not be interpreted as evidence that all forms of mechanical stimulation are protective. In a murine post-traumatic OA model, exercise intensity and αCGRP signalling modified cartilage extracellular-matrix stiffness, inflammatory responses and subchondral or metaphyseal bone remodelling, whereas gross cartilage degradation was not substantially altered by exercise intensity (Pann et al., 2025). In bone, non-enzymatic glycation cross-linking can modify osteocyte–matrix interactions and mechanosensitivity. Moderate changes in matrix cross-linking may alter force transmission, whereas excessive accumulation of glycation-associated cross-links can reduce matrix quality and impair cellular mechanoadaptation (Liu et al., 2025). This bone-specific mechanism should be distinguished from cartilage- and disc-specific matrix-remodelling processes. Abnormal mechanical loading, inflammatory signalling, mitochondrial stress and matrix degradation form mutually reinforcing processes in OA and IVDD. Cell death may contribute to this network by reducing the number of matrix-maintaining cells and releasing inflammatory signals, but mechanical dysregulation and extracellular matrix loss should not automatically be classified as direct molecular crosstalk among death programmes. Representative examples of how cell-death-associated events remodel disease-specific skeletal microenvironments, including cartilage, bone-remodelling and intervertebral-disc compartments, are summarized in Table 2.

**TABLE 2:** Cell-death-associated remodelling of disease-specific skeletal microenvironments.

Disease/anatomical compartment	Principal cell or model	Death-associated event	Key molecular axis	Tissue-level consequence	Evidence interpretation	Principal limitation	Ref.
osteoarthritis (OA)/articular cartilage	Chondrocytes exposed to inflammatory stress	Reduced autophagy-associated activity and increased apoptosis	sirtuin 3(SIRT3)–phosphoinositide 3-kinase (PI3K)–protein kinase B (AKT)–mechanistic target of rapamycin (mTOR)	Loss of matrix-maintaining cells and cartilage homeostasis	Shared upstream regulation of autophagy and apoptotic susceptibility	Direct interaction between autophagic and apoptotic execution mechanisms was not established	(Xu et al., 2021)
OA/articular cartilage	tert-butyl hydroperoxide (TBHP)-stressed chondrocytes and experimental OA	Oxidative- and endoplasmic reticulum (ER)-stress-associated apoptosis	AMP-activated protein kinase (AMPK–SIRT3); peroxisome proliferator-activated receptor-γ coactivator 1α(PGC-1α); mitochondrial transcription factor A (TFAM); nuclear factor erythroid 2-related factor 2(NRF2)	Mitochondrial dysfunction, reduced type II collagen (COL2) and aggrecan, increased matrix metalloproteinase 13 (MMP13) and structural damage	Organelle-stress regulation of apoptosis and matrix catabolism	Evidence remains predominantly preclinical, and its temporal relevance to human OA is uncertain	(Qiao et al., 2025)
OA/articular cartilage	interleukin-1 beta (IL-1β)-stimulated chondrocytes	Mitophagy-associated changes, apoptosis and inflammasome-associated inflammation	arachidonate 5-lipoxygenase (ALOX5)–nuclear factor-κB (NF-κB)–NLR family pyrin domain containing 3(NLRP3)	Inflammatory amplification and matrix degradation	Shared upstream regulation of mitochondrial quality control and inflammatory injury	Gasdermin-dependent pyroptotic execution and direct pathway dependency were not demonstrated	(Zhou et al., 2025b)
OA/articular cartilage	Chondrocytes with altered integrin subunit beta 1(ITGB1) signalling	Apoptosis-associated loss of viability	ITGB1–cyclic adenosine monophosphate (cAMP)–protein kinase A (PKA)	Reduced chondrocyte survival and cartilage integrity	Integrin-associated survival and inflammatory regulation of apoptosis	The study did not directly examine mechanical loading or crosstalk between distinct death programmes	(Xie et al., 2023b)
osteoporosis (OP)/osteoblast compartment	Osteoblasts with dysregulated iron metabolism	Mitochondrial apoptosis	replication initiator 1 (REPIN1)–lipocalin 2 (LCN2); BCL-2-associated X protein (BAX)/B-cell lymphoma 2(BCL2)–cytochrome c–caspase-3(CASP3)	Impaired osteogenic differentiation and mineralization	Iron-metabolism-associated regulation of intrinsic apoptosis	The contribution of osteoblast apoptosis to whole-bone loss requires lineage-specific in vivo validation	(Xia et al., 2023c)
OP/osteoblast compartment	Osteoblasts, ovariectomy (OVX)-induced OP bone and human OP specimens	Ferroptosis	DNA methyltransferase 1 (DNMT1)/DNA methyltransferase 3A (DNMT3A)/DNA methyltransferase 3B (DNMT3B)–glutathione peroxidase 4 (GPX4)-promoter methylation	Reduced osteogenic markers and aggravated bone loss	Epigenetic regulation of ferroptotic susceptibility	Mechanistic specificity depends on methylation-specific manipulation and ferroptosis-directed rescue; clinical evidence remains limited	(Ruan et al., 2024)
Diabetic OP/osteocyte network	Osteocytes under diabetic glucolipotoxic stress	Ferroptosis	NRF2–c-JUN–haem oxygenase 1(HO-1)	Osteocyte loss, empty lacunae and impaired bone formation	Lineage-specific ferroptotic injury affecting bone-remodelling signals	NRF2/HO-1 effects are context-dependent, and translation from diabetic animal models remains uncertain	(Yang et al., 2022a)
Age-related OP/osteoblast compartment	Senescent osteoblasts	Ferroptosis accompanied by senescence-associated changes	vitamin D receptor (VDR)–NRF2–GPX4	Reduced osteoblast function and bone formation	Shared redox regulation of ferroptosis and senescence	Bidirectional causal dependence between ferroptosis and senescence was not established	(Xu et al., 2022)
OP/osteoclast compartment	receptor activator of NF-κB ligand (RANKL)-stimulated osteoclast-lineage cells	Ferroptosis-associated changes during differentiation	NF-κB; GPX4; acyl-CoA synthetase long-chain family member 4 (ACSL4)	Altered osteoclastogenesis and bone resorption	Cell-lineage-dependent ferroptotic regulation of bone remodelling	The direction of ferroptosis-related effects in osteoclasts may differ among models and requires pathway-specific rescue	(Xu et al., 2023c)
OP/osteoblast compartment	Osteoblasts under oxidative and iron-related stress	Ferroptosis	Kelch-like ECH-associated protein 1(KEAP1)–NRF2–solute carrier family 7 member 11(SLC7A11)–GPX4	Impaired osteogenesis and bone loss	Antioxidant-pathway regulation of ferroptotic susceptibility	Mangiferin is pleiotropic; target dependency should be distinguished from general antioxidant effects	(Deng et al., 2024)
Postmenopausal OP/bone marrow mesenchymal stem cell (BMSC) compartment	Human BMSCs and OVX-related models	Ferroptosis during impaired osteogenic differentiation	SLC7A11/solute carrier family 3 member 2(SLC3A2); bone morphogenetic protein 2(BMP2)–small mothers against decapentaplegic (SMAD)	Reduced osteogenesis and deteriorated trabecular microarchitecture	Parallel regulation of ferroptosis and osteogenic signalling	Direct ferroptosis–osteogenesis dependency requires pathway-specific perturbation and rescue	(Zheng et al., 2025)
Postmenopausal OP/bone-remodelling network	circular RNA (circRNA)–microRNA (miRNA)–messenger RNA (mRNA) network analysis	Ferroptosis-associated molecular signature	circRNA/miR-23b-3p/phosphatase and tensin homolog (PTEN); PI3K–AKT	Predicted effects on proliferation, metabolism, inflammation and angiogenesis	Hypothesis-generating network association	Computational associations should not be presented as established causal mechanisms or therapeutic efficacy	(Huang et al., 2024)
OA/cartilage and subchondral-bone compartments	IL-1β-stimulated chondrocytes and RANKL-stimulated osteoclasts	Pyroptosis-associated cartilage injury and osteoclastogenesis	NF-κB–NLRP3–caspase-1(CASP1); RANKL–nuclear factor of activated T cells 1(NFATC1)	Matrix degradation and abnormal subchondral-bone remodelling	Parallel effects on chondrocyte pyroptosis and osteoclastogenesis	Effects in two cell populations do not establish direct intercompartmental communication or pyroptosis–osteoclastogenesis crosstalk	(Zheng et al., 2023)
OA/articular cartilage	IL-1β-stimulated chondrocytes	Pyroptosis-associated injury	PI3K–AKT–NF-κB–NLRP3–CASP1–gasdermin D (GSDMD)	Inflammation, extracellular matrix (ECM) degradation and chondrocyte loss	Inflammasome- and gasdermin-associated regulation of matrix homeostasis	Upstream pathway inhibition is pleiotropic; lytic execution should be verified using gasdermin-dependent rescue	(Liu et al., 2023a)
OA/articular cartilage	Chondrocytes with mitochondrial stress	Pyroptosis-associated injury	NRF2-associated mitochondrial regulation; NLRP3–CASP1–GSDMD	Mitochondrial dysfunction, inflammatory amplification and ECM loss	Redox and mitochondrial regulation of inflammasome-associated injury	The study supports shared upstream regulation but does not establish crosstalk with other death programmes	(Li et al., 2025b)
OA/synovium–cartilage unit	Synovial macrophages and neighbouring chondrocytes	Macrophage pyroptosis-associated inflammation	Mitochondrial reactive oxygen species (ROS)–NF-κB–NLRP3	Synovial inflammation and secondary chondrocyte matrix injury	Intercellular propagation of inflammatory signals	Macrophage-state heterogeneity and validation in human OA synovium remain limited	(Ma et al., 2025)
intervertebral disc degeneration (IVDD)/nucleus pulposus compartment	nucleus pulposus (NP) cells under oxidative and inflammatory stress	Concurrent pyroptosis- and ferroptosis-associated changes	NRF2 deubiquitination and antioxidant defence	Reduced NP-cell injury and ECM degradation after intervention	Coordinated regulation through a shared antioxidant node	Simultaneous suppression of two marker sets does not prove direct pyroptosis–ferroptosis crosstalk	(Wu et al., 2024)
IVDD/cartilage endplate	Cartilage-endplate chondrocytes under oxidative and calcifying stress	Apoptosis-associated injury	PTEN–PI3K–AKT–NRF2	Reduced apoptosis, calcification and ECM deterioration; preservation of cartilage-endplate integrity	Upstream redox and survival-pathway regulation of apoptosis	The study did not establish ferroptosis or mitophagy dependency; evidence remains preclinical	(Cui et al., 2023)

Abbreviations: ACSL4, acyl-CoA, synthetase long-chain family member 4; AKT, protein kinase B; ALOX5, arachidonate 5-lipoxygenase; AMPK, AMP-activated protein kinase; BAX, BCL-2-associated X protein; BCL2, B-cell lymphoma 2; BMP2, bone morphogenetic protein 2; BMSC, bone marrow mesenchymal stem cell; cAMP, cyclic adenosine monophosphate; CASP1, caspase-1; CASP3, caspase-3; circRNA, circular RNA; COL2, type II, collagen; DNMT1, DNA, methyltransferase 1; DNMT3A, DNA, methyltransferase 3A; DNMT3B, DNA, methyltransferase 3B; ECM, extracellular matrix; ER, endoplasmic reticulum; GPX4, glutathione peroxidase 4; GSDMD, gasdermin D; HO-1, haem oxygenase 1; IL-1β, interleukin-1, beta; ITGB1, integrin subunit beta 1; IVDD, intervertebral disc degeneration; KEAP1, Kelch-like ECH-associated protein 1; LCN2, lipocalin 2; miRNA, microRNA; MMP13, matrix metalloproteinase 13; mRNA, messenger RNA; mTOR, mechanistic target of rapamycin; NFATc1, nuclear factor of activated T cells 1; NF-κB, nuclear factor-κB; NLRP3, NLR, family pyrin domain containing 3; NP, nucleus pulposus; NRF2, nuclear factor erythroid 2-related factor 2; OA, osteoarthritis; OP, osteoporosis; OVX, ovariectomy; PGC-1α, peroxisome proliferator-activated receptor-γ coactivator 1α; PI3K, phosphoinositide 3-kinase; PKA, protein kinase A; PTEN, phosphatase and tensin homolog; RANKL, receptor activator of NF-κB, ligand; REPIN1, replication initiator 1; ROS, reactive oxygen species; SIRT3, sirtuin 3; SLC3A2, solute carrier family 3 member 2; SLC7A11, solute carrier family 7 member 11; SMAD, small mothers against decapentaplegic; TBHP, tert-butyl hydroperoxide; TFAM, mitochondrial transcription factor A; VDR, vitamin D receptor.

## 5 Epigenetic, redox and metabolic regulation of cell-death susceptibility

**FIGURE 4:**
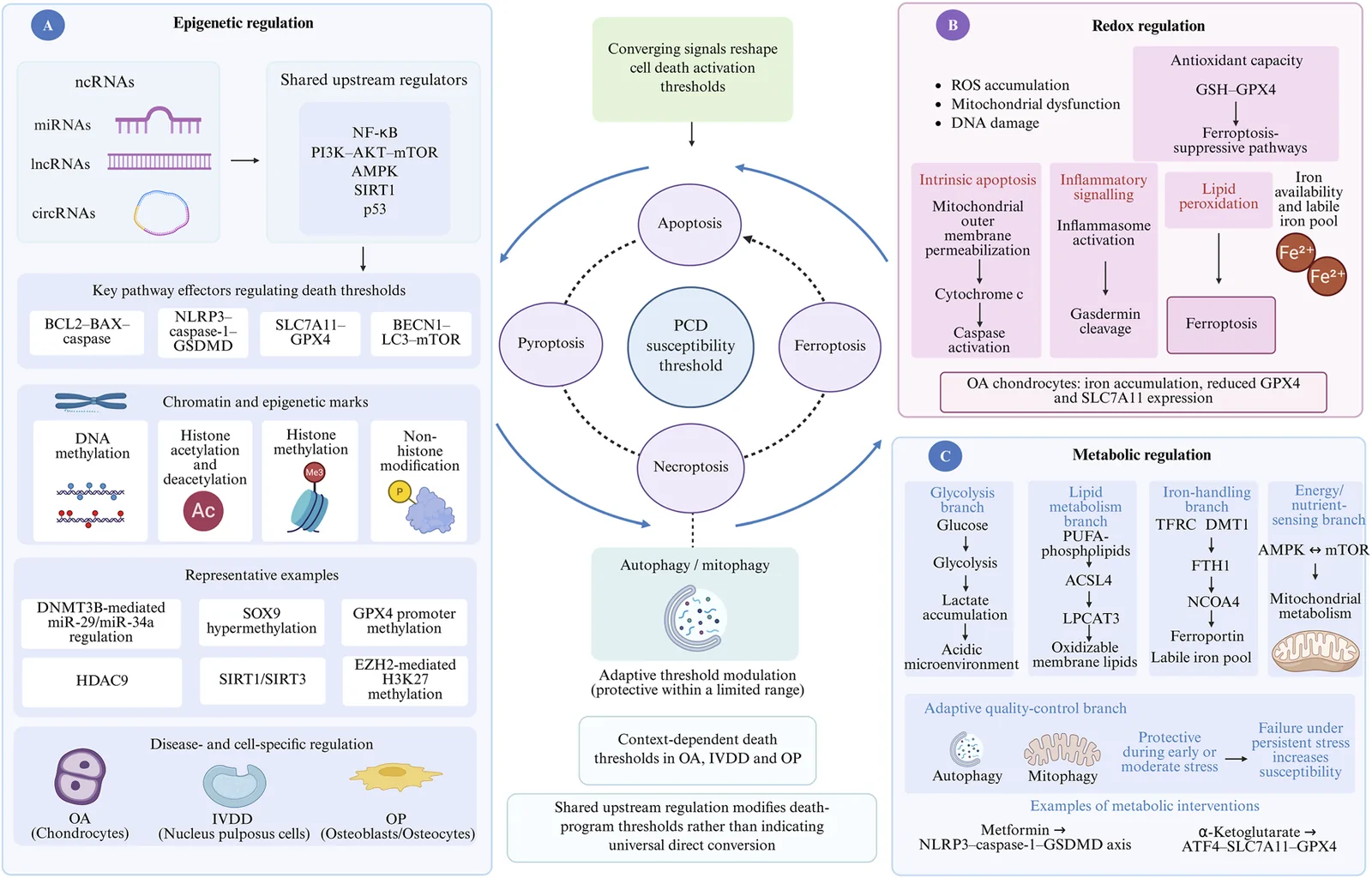
Epigenetic, redox and metabolic regulation of cell-death susceptibility in degenerative skeletal diseases. **(A)** Non-coding RNAs and epigenetic modifications regulate cell-death susceptibility through shared upstream nodes, including NF-κB, PI3K–AKT–mTOR, AMPK, SIRT1 and p53, and downstream effectors associated with apoptosis, pyroptosis, ferroptosis and autophagy. DNA methylation, histone acetylation or deacetylation, histone methylation and non-histone protein modifications reshape stress-responsive transcription in a disease-, cell- and context-dependent manner. **(B)** Redox imbalance increases mitochondrial dysfunction, DNA damage, inflammasome activation, lipid peroxidation and labile iron availability. The resulting outcome depends on mitochondrial integrity, gasdermin activation, iron availability and ferroptosis-suppressive systems, particularly the GSH–GPX4 axis. **(C)** Metabolic regulation modifies cell-death thresholds through glycolysis and lactate accumulation, ACSL4–LPCAT3-dependent phospholipid remodelling, iron handling and AMPK–mTOR-dependent nutrient sensing. Autophagy and mitophagy generally support adaptation during early or moderate stress, whereas failure or dysregulation of these quality-control processes under persistent stress increases susceptibility to cell injury and death. Metabolic interventions, including metformin- and α-ketoglutarate-associated regulation, may therefore affect different death-associated pathways through distinct upstream mechanisms. Collectively, epigenetic, redox and metabolic signals converge on context-dependent susceptibility thresholds for apoptosis, pyroptosis, necroptosis and ferroptosis. Abbreviations: ACSL4, acyl-CoA synthetase long-chain family member 4; AKT, protein kinase B; AMPK, AMP-activated protein kinase; ATF4, activating transcription factor 4; BAX, BCL-2-associated X protein; BECN1, beclin 1; circRNA, circular RNA; DMT1, divalent metal transporter 1; FTH1, ferritin heavy chain 1; GPX4, glutathione peroxidase 4; GSH, glutathione; GSDMD, gasdermin D; IVDD, intervertebral disc degeneration; lncRNA, long non-coding RNA; LPCAT3, lysophosphatidylcholine acyltransferase 3; miRNA, microRNA; mTOR, mechanistic target of rapamycin; ncRNA, non-coding RNA; NCOA4, nuclear receptor coactivator 4; NF-κB, nuclear factor-κB; NLRP3, NLR family pyrin domain containing 3; OA, osteoarthritis; OP, osteoporosis; PCD, programmed cell death; PI3K, phosphoinositide 3-kinase; ROS, reactive oxygen species; SIRT, sirtuin; SLC7A11, solute carrier family 7 member 11; TFRC, transferrin receptor.

In degenerative skeletal diseases, cell-death programmes are regulated by interconnected transcriptional, epigenetic, redox and metabolic networks rather than by isolated linear pathways. Non-coding RNAs, chromatin modifications, mitochondrial function, iron handling and cellular metabolism can alter the activation thresholds of apoptosis, pyroptosis, necroptosis and ferroptosis. However, simultaneous regulation of several pathways should generally be interpreted as shared upstream control unless direct dependency between their execution machineries has been demonstrated. As summarized in Figure 4, non-coding RNAs and chromatin modifications, redox balance, mitochondrial quality control, iron handling and metabolic state converge to modify the susceptibility thresholds of multiple cell-death programmes. These regulatory networks may influence several death-associated pathways simultaneously, but such convergence generally reflects shared upstream control rather than direct interaction between their execution machineries.

### 5.1 Non-coding RNA regulation of cell-death-associated pathways

A large proportion of the human genome does not encode conventional proteins, and a proportion of this non-coding genome gives rise to regulatory RNAs, including miRNAs, lncRNAs and circRNAs (Zheng Y. et al., 2021). MiRNAs commonly regulate gene expression through sequence-dependent interaction with target transcripts. LncRNAs and circRNAs can act through diverse mechanisms, including transcriptional regulation, protein interaction, RNA stability and, in selected cases, competing endogenous RNA networks (Djuranovic et al., 2012; Valin et al., 2014; Mathy and Chen, 2017; Zhang J. et al., 2024; Panda, 2018; Jarlstad Olesen and Kristensen, 2021). A ceRNA or miRNA-sponge mechanism should be concluded only when physical interaction, appropriate subcellular localization, sufficient transcript abundance and functional perturbation or rescue are demonstrated. Accumulating evidence indicates that ncRNAs regulate apoptosis-, pyroptosis-, ferroptosis- and autophagy-associated pathways. NcRNAs regulate apoptosis-associated BCL2–BAX signalling (Yan et al., 2016; Wang et al., 2019; Zhao et al., 2017), pyroptosis-associated inflammasome signalling (Zhang et al., 2024) and ferroptosis-associated SLC7A11–GPX4 regulation (Yan et al., 2016). Evidence for ncRNA regulation of autophagy should be discussed separately using pathway-specific studies. In OA, miR-34a increases chondrocyte apoptotic susceptibility through SIRT1–p53-associated regulation (Yan et al., 2016). These findings indicate that ncRNAs can modify apoptotic susceptibility, but they do not demonstrate interaction between apoptosis and other death-execution pathways. In IVDD, miR-222 promotes nucleus pulposus-cell apoptosis through BCL2-associated regulation (Wang et al., 2019). Hypomethylation and increased expression of miR-143 have also been associated with suppression of anti-apoptotic signalling and reduced nucleus pulposus-cell survival (Zhao et al., 2017). In OP-related models, lncRNA GM15416 reduces osteoblast apoptosis through cellular Fos proto-oncogene (c-Fos)–Fas-associated regulation (Zhao et al., 2024), whereas circZfp644-205 regulates the miR-455-3p–SMAD2 axis and impairs osteogenic differentiation (Zhang P. et al., 2024). These observations support disease- and cell-specific ncRNA regulation rather than a uniform ncRNA network across OA, IVDD and OP.

NcRNAs also regulate inflammasome- and pyroptosis-associated responses. In OA, the miR-124-3p–MALAT1–KLF5–CXCL11 network has been linked to reduced chondrocyte pyroptosis and cartilage injury (Rozi et al., 2022). In IVDD, miRNA-141 promotes ROS generation and TXNIP–NLRP3-associated pyroptotic injury in nucleus pulposus cells (Xu Q. et al., 2020). Autophagy and NFE2L2 provide additional, non-ncRNA-mediated protection against oxidative-stress-associated pyroptosis (Bai et al., 2020). In osteoblast injury models, the miR-18a–HIF-1α axis is associated with NLRP3 activation and concurrent changes in several death-associated markers (Zhang et al., 2024). These studies support upstream regulation of inflammatory cell-death susceptibility but do not necessarily establish direct crosstalk among execution programmes.

NcRNA regulation of ferroptosis is concentrated around iron metabolism, phospholipid remodelling and antioxidant defence. In OA, miR-181b and synovial-fibroblast-derived exosomal miR-19b-3p have been reported to suppress SLC7A11 and increase chondrocyte ferroptotic susceptibility (Wang et al., 2023; Kong et al., 2023). Conversely, lncRNA MEG3 regulates the miR-885-5p–SLC7A11 axis and reduces ferroptosis-associated injury (Zhu et al., 2024). These examples suggest that both intracellular and extracellular-vesicle-associated ncRNAs can modify chondrocyte redox homeostasis. In IVDD, BACH1-mediated regulation of the HMOX1–GPX4 axis and the circ-STC2–miR-486-3p–TFR2 pathway have been associated with ferroptotic nucleus pulposus-cell injury (Yao et al., 2023; Xiong et al., 2023). Nucleus-pulposus-progenitor-cell-derived exosomes may increase resistance to ferroptosis through transfer of miR-221–3p (Wang W. et al., 2025). In a steroid-induced osteonecrosis model, knockdown of lncRNA XR_877,193.1 reduced dexamethasone-associated ferroptosis and improved osteogenic differentiation in MC3T3-E1 cells (Yang et al., 2025). By contrast, circRNA–miRNA–mRNA networks identified only through computational analysis should be regarded as hypotheses until experimentally validated (Li H. et al., 2022). Overall, ncRNAs can regulate several death-associated pathways through shared upstream hubs such as NF-κB, PI3K–AKT–mTOR, AMPK, SIRT1 and p53. Such network-level regulation may alter cellular susceptibility to multiple death programmes, but it should not be described as direct molecular crosstalk unless pathway-specific interdependence is demonstrated.

### 5.2 Epigenetic regulation of cell-death-associated gene expression

Epigenetic modifications influence chromatin accessibility and transcriptional responses without altering the underlying DNA sequence. DNA methylation, histone acetylation, histone methylation and non-histone protein modifications can alter the expression of genes involved in inflammation, mitochondrial function, antioxidant defence and extracellular matrix homeostasis (Ruan et al., 2024; Chen J. et al., 2024; Yao et al., 2025; Luo et al., 2025; Hao et al., 2022). Their effects are gene-, cell- and context-dependent and should not be interpreted as uniformly activating or repressing cell death. In OA and IVDD, differential DNA methylation has been identified in genes associated with inflammation, metabolism, extracellular matrix turnover and cell survival (Hou et al., 2026; Shen et al., 2017). Promoter hypomethylation of MMP3, MMP13, IL1B and IL8 has been associated with increased catabolic or inflammatory gene expression, whereas hypermethylation of SOX9 can impair chondrocyte anabolic capacity (Shen et al., 2017; Ball et al., 2022). These changes may lower cellular tolerance to inflammatory and mechanical stress but do not independently establish activation of a particular death programme.

DNA methylation can also interact with ncRNA networks. Epigenetic regulation of miR-29 and miR-34a has been implicated in cartilage metabolism and chondrocyte apoptosis (Veronesi et al., 2023). LncRNA SNHG9 has been reported to regulate miR-34a methylation and reduce chondrocyte apoptosis (Zhang et al., 2020). In IVDD, hypomethylation of the miR-143 promoter is associated with increased miR-143 expression and impaired anti-apoptotic signalling (Zhao et al., 2017). These findings should be interpreted within the specific cell and experimental models in which they were demonstrated. Epigenetic regulation may additionally influence ferroptotic susceptibility. In OP models, increased DNA methyltransferase (DNMT)1, DNMT3A and DNMT3B expression promotes GPX4 promoter hypermethylation, while KLF5 and the transcriptional corepressors NCoR and SnoN contribute to GPX4 transcriptional repression, thereby increasing osteoblast ferroptotic susceptibility (Ruan et al., 2024). Pharmacological inhibition of KLF5 partially restores GPX4 expression, although the specificity and translational relevance of this intervention require further validation.

Histone acetylation and deacetylation regulate transcriptional accessibility and stress-responsive gene expression. HDAC9 has been implicated in inflammation, apoptosis and matrix catabolism in nucleus pulposus cells (Lei et al., 2023). HDAC-associated regulation of RUNX2 and MMP13 may also influence chondrocyte differentiation and matrix turnover (Cai et al., 2022). Sirtuins connect metabolic state with protein acetylation, mitochondrial function and stress responses. SIRT1 regulates p53-and NF-κB-associated signalling, whereas SIRT3 primarily modifies mitochondrial proteins and redox homeostasis. Their actions may alter apoptotic or ferroptotic susceptibility, but they should not automatically be classified as direct regulation of death-mode conversion (Liu Y. et al., 2023; Xu M. et al., 2020). Histone methylation can generate activating or repressive chromatin states depending on the residue and methylation context. The EZH2–miR-142-3p–HMGB1 axis links epigenetic regulation and ER stress to chondrocyte pyroptosis and cartilage injury in experimental OA (Chen Y. et al., 2025). Regulation of the H3K36 methyltransferase NSD1 by macrophage-derived miRNAs may further modify chondrocyte transcriptional programmes (Lin R. et al., 2024). In contrast, EZH2-mediated methylation of DDX1 at lysine 234 represents a non-histone protein modification and should be distinguished from histone methylation (ZHU et al., 2025c). Epigenetic modifications reshape the transcriptional context in which cell-death programmes are activated. Most available studies demonstrate altered susceptibility or shared upstream regulation rather than direct interconversion among apoptosis, pyroptosis and ferroptosis.

### 5.3 Oxidative stress and metabolic reprogramming as regulators of cell-death susceptibility

Reactive oxygen species have physiological signalling functions at controlled concentrations, but excessive or persistent ROS production can damage mitochondrial components, DNA and membrane lipids and can activate inflammatory pathways. The resulting cell-fate outcome depends on antioxidant capacity, mitochondrial integrity, iron availability, membrane lipid composition, energy status and the intensity and duration of stress (Miao et al., 2022; Xia S. et al., 2025; Sheng et al., 2024). Oxidative stress should therefore be viewed as a shared upstream regulator of cell-death susceptibility rather than as a determinant that invariably selects one execution programme. Mitochondrial outer-membrane permeabilization, cytochrome-c release and caspase activation favour intrinsic apoptosis. Inflammasome activation and gasdermin cleavage can promote pyroptotic membrane permeabilization. By contrast, iron-dependent oxidation of susceptible phospholipids, together with failure of the GSH–GPX4 or other ferroptosis-suppressive systems, promotes ferroptosis. These processes may occur independently or in parallel, and their relative contribution must be established by pathway-specific perturbation. In OA, iron accumulation, lipid-peroxidation-associated damage and reduced GPX4 or SLC7A11 expression have been reported in chondrocytes (Miao et al., 2022; Yao et al., 2021). However, mitochondrial shrinkage and changes in individual ferroptosis-associated proteins are not sufficient on their own to establish ferroptotic death. Functional rescue using ferroptosis-directed inhibitors or genetic manipulation provides stronger evidence.

Metabolic reprogramming can further modify cell-death susceptibility. Nucleus pulposus cells depend substantially on glycolysis because of the hypoxic and poorly vascularized disc environment. Excessive lactate accumulation and extracellular acidification may impair matrix homeostasis and increase inflammatory or apoptotic stress (Wu et al., 2021). Nevertheless, glycolysis is also required for physiological nucleus pulposus-cell survival, and its effects should not be considered uniformly harmful. Lactate may influence ferroptotic susceptibility through context-dependent mechanisms, including regulation of gene expression and protein lactylation, but it should not be described as a universal ferroptosis-inducing metabolite (Sun K. et al., 2025). Evidence obtained in one cell type or disease model should not be directly extrapolated to OA, IVDD and OP as a whole. Lipid metabolism has a more direct role in ferroptosis. ACSL4-and LPCAT3-dependent incorporation of polyunsaturated fatty acids into membrane phospholipids can increase the pool of oxidizable lipid substrates. Iron-handling proteins, including TFRC, DMT1, FTH1, NCOA4 and ferroportin, regulate the labile iron pool and thereby influence lipid-peroxidation reactions. However, the effect of each regulator remains dependent on cell lineage and disease context (Miao et al., 2022; Sheng et al., 2024). During early or moderate stress, autophagy and mitophagy may preserve organelle quality and limit ROS accumulation. Under persistent or severe stress, failure or dysregulation of these adaptive processes can increase susceptibility to apoptosis, pyroptosis or ferroptosis. This represents a change in the activation threshold of individual death programmes rather than a universal sequential conversion from one form of cell death to another.

Metformin-mediated suppression of NLRP3–caspase-1–GSDMD-associated responses and α-ketoglutarate-mediated regulation of the ETV4–SLC7A11–GPX4 pathway illustrate that distinct metabolic interventions can influence different death-associated pathways (Yan et al., 2022; He R. et al., 2024). However, these independent observations do not establish direct molecular crosstalk or conversion between pyroptosis and ferroptosis. Overall, oxidative stress and metabolic reprogramming establish a context-dependent regulatory environment that modifies the thresholds of several cell-death programmes. Their effects depend on cell identity, anatomical niche, disease stage and the availability of specific execution and defence mechanisms.

## 6 Preclinical therapeutic strategies targeting cell-death networks and barriers to clinical translation

### 6.1 Multi-node and combinatorial interventions

Cell-death-associated responses in degenerative skeletal diseases are shaped by interconnected inflammatory, oxidative, mitochondrial and metabolic disturbances. This convergence provides a rationale for modulating more than one pathogenic process. However, simultaneous changes in several molecular markers do not demonstrate therapeutic synergy or direct crosstalk among death programmes. A single compound may exert pleiotropic effects through a shared upstream regulator, whereas a genuine combinatorial strategy requires evidence that two or more mechanistically distinct interventions provide additive or synergistic benefit beyond appropriately optimized monotherapies. Most current evidence remains preclinical, and direct comparisons between multi-node interventions, combination regimens and single-target controls remain limited.

In pyroptosis-associated injury, experimental interventions commonly combine suppression of inflammatory signalling with preservation of mitochondrial quality control. MiR-155 knockdown reduces chondrocyte pyroptosis-associated responses through SMAD2-related regulation and inhibition of the NLRP3–caspase-1 pathway (Li G. et al., 2022). AZ similarly attenuates pyroptosis while promoting PINK1–Parkin-dependent mitophagy (Zhang Z. et al., 2025). These observations suggest that inflammasome regulation and mitochondrial quality control may converge to reduce inflammatory cell injury. However, because these interventions affect broad upstream stress networks, their effects are more appropriately interpreted as multi-node regulation rather than direct mitophagy–pyroptosis crosstalk.

A similar principle applies to ferroptosis-oriented interventions. AZ activates Nrf2–HO-1-associated antioxidant signalling and reduces IL-1β-induced ferroptotic injury (Chen X. et al., 2023). In IVDD models, hesperidin enhances Nrf2-associated antioxidant defence and suppresses NF-κB-associated inflammatory signalling, thereby reducing oxidative-stress-induced nucleus pulposus-cell ferroptosis (Zhu et al., 2023). Vitamin K2 increases GPX4-associated antioxidant capacity and inhibits MAPK–NF-κB signalling, thereby reducing lipid peroxidation, inflammation and extracellular matrix degradation (He Q. et al., 2024). These findings support the therapeutic potential of shared redox and inflammatory regulatory nodes. Nevertheless, simultaneous modulation of several pathways may reflect the pleiotropic pharmacology of an individual compound rather than synergy between independently targeted mechanisms.

Autophagy-associated interventions have also been investigated as approaches to limiting apoptosis and matrix catabolism. Methyl gallate enhances SIRT3-mediated autophagy and reduces apoptosis- and extracellular-matrix-degradation-associated responses (Li Y. et al., 2023). Vitamin D modulates AMPK–mTOR-associated autophagy while reducing chondrocyte apoptosis and extracellular matrix loss (Liu P. et al., 2024), whereas temsirolimus promotes autophagy-associated protection in experimental OA (Otsuki et al., 2025). These findings support restoration of autophagic quality control under selected conditions. They should not, however, be generalized into a universal model in which autophagy activation invariably suppresses apoptosis. The consequences of autophagy depend on autophagic flux, cargo selectivity, cell identity and disease stage, and selective pathways such as ferritinophagy may increase ferroptotic susceptibility.

Shared regulators such as ROS, NF-κB, Nrf2, mitochondrial dysfunction and metabolic stress may provide context-dependent entry points for modulating apoptosis-, pyroptosis-, ferroptosis- and autophagy-associated responses (Tao et al., 2024; Wu et al., 2023; Fang et al., 2023). However, targeting a central hub may also disrupt adaptive or reparative responses. The current evidence therefore supports further evaluation of context-specific multi-node strategies but does not establish their superiority over well-selected single-target interventions. Future studies should directly compare monotherapy and combination arms, quantify additive or synergistic effects, define treatment sequence and therapeutic windows, and determine whether benefit is sustained without excessive inhibition of physiological stress adaptation or tissue repair.

### 6.2 Local and engineered delivery systems: opportunities and limitations

Local and engineered delivery systems are being developed to increase tissue exposure to cell-death-modulating agents while reducing systemic exposure. These approaches may be particularly relevant to articular cartilage, intervertebral discs and bone, where dense extracellular matrices, limited vascularity and complex mechanical environments restrict penetration and retention. However, tissue accumulation in small-animal models should not be equated with clinically meaningful targeting. Differences in tissue dimensions, vascular supply, matrix composition, clearance and mechanical loading may substantially alter delivery performance in humans. Claims of targeted delivery should therefore be supported by quantitative biodistribution, tissue retention, cargo-release kinetics, target engagement and biological activity.

Extracellular vesicles are frequently investigated as carriers of proteins, nucleic acids and regulatory RNAs. S-EXO-loaded hydrogel microspheres deliver superoxide dismutase (SOD)3 and reduce oxidative stress in experimental OA (Cao et al., 2024). Human amniotic epithelial stem-cell-derived extracellular vesicles transfer lncRNA ACTA2-AS1 and promote ACSL4 degradation, thereby reducing chondrocyte ferroptosis-associated injury (Wang X. et al., 2025). BMSC-derived extracellular vesicles have also been reported to suppress METTL3 and modulate the METTL3–m6A–ACSL4 axis (Cheng et al., 2024). These studies illustrate the ability of extracellular vesicles to combine cargo delivery with intrinsic biological signalling. Nevertheless, their therapeutic activity cannot automatically be attributed to targeting, because vesicle cargo composition, membrane components, uptake efficiency and endogenous vesicle activity may each contribute to the observed effects.

Extracellular-vesicle-mediated miRNA delivery has also been used to regulate inflammation and apoptosis. Human umbilical-cord-MSC-derived vesicles transfer miR-199a-3p, suppress MAPK4–NF-κB signalling and reduce inflammatory and apoptotic responses in chondrocytes (Chen L-Q. et al., 2025). Vesicles derived from quercetin-preconditioned BMSCs deliver miR-124–3p and regulate TRAF6-, p38 MAPK- and NF-κB-associated signalling (Dong et al., 2024). Although these studies demonstrate functional cargo transfer, comparisons with free RNA, unmodified vesicles, cargo-depleted vesicles, non-targeted carriers and conventional delivery systems are required to establish the contribution of the carrier and any delivery advantage.

Stimuli-responsive nanoparticles provide an additional approach to locally controlled cargo release. ROS-responsive systems can increase cargo release under oxidatively stressed experimental conditions and modulate macrophage polarization, mitochondrial function and inflammatory signalling (Li H. et al., 2024). GelMA@APPA microspheres show effective chondrocyte internalization, improve chondrocyte proliferation and reduce apoptosis-associated changes in experimental OA (Wang J. et al., 2025). However, responsiveness demonstrated under simplified experimental conditions may not predict release kinetics in heterogeneous human tissues, where ROS levels, pH, enzymatic activity and mechanical forces vary spatially and temporally.

Before clinical translation, these platforms require standardized physicochemical characterization, appropriate empty-carrier and non-targeted controls, quantitative pharmacokinetic and biodistribution studies, repeated-dose evaluation and assessment of local and systemic toxicity. Extracellular-vesicle studies require standardized characterization, dose reporting, purity assessment, storage conditions and functional controls in accordance with current EV-reporting recommendations (Welsh et al., 2024). Current delivery platforms should therefore be regarded as experimental approaches for improving local modulation of cell-death-associated pathways rather than established precision therapies.

### 6.3 Biomaterials and tissue-engineering approaches

Biomaterials and tissue-engineering approaches may integrate local cargo delivery, mechanical support and reconstruction of the tissue microenvironment. This is potentially relevant to degenerative skeletal diseases, in which molecular injury and structural deterioration occur concurrently. However, effects arising from cell-death-pathway modulation must be distinguished from those caused by altered matrix stiffness, cell adhesion, load distribution or endogenous tissue repair.

An injectable thermosensitive hydrogel containing ROS-responsive dihydroartemisinin-prodrug-loaded Soluplus/TPGS nanomicelles (DHA-PRO@NMs@TSH) reduced apoptosis-, necroptosis- and pyroptosis-associated markers in experimental chondrocytes and attenuated OA-related cartilage injury (Li et al., 2026). A platinum-nanozyme-loaded silk fibroin/oxidized pullulan hydrogel (Pt@SF/oxPL) reduced reactive oxygen species accumulation and ferroptosis-associated chondrocyte injury in experimental OA (Feng J. et al., 2024).

Dihydromyricetin-loaded methacrylated hyaluronic acid–gelatin methacryloyl–benzenediboronic acid hydrogel microspheres (DMY@HAMA–GelMA–PBA; DMY@HGP) modulated SIRT3-associated mitophagy and mitochondrial apoptosis in experimental OA (Xia X. et al., 2023). p-Synephrine-loaded injectable GelMA hydrogel suppresses chondrocyte apoptosis and attenuates cartilage degeneration in experimental OA (Yu Y. et al., 2025). These systems demonstrate the feasibility of combining controlled release with tissue support. Nevertheless, the respective contributions of the biomaterial, incorporated cargo and altered mechanical environment should be evaluated using unloaded-material, free-cargo and matched-physical-property controls.

Material properties may also influence cell fate independently of the incorporated therapeutic agent. Stiffness, porosity, degradation rate, surface chemistry and cell-adhesion characteristics can modify mechanotransduction, inflammatory signalling, mitochondrial function and lineage differentiation. Reductions in cell-death-associated markers may therefore result from improved mechanical compatibility or altered cellular phenotype rather than direct inhibition of a death-execution pathway.

Translational evaluation must extend beyond short-term biocompatibility and histological improvement. Long-term studies should examine structural integration, mechanical durability, chronic inflammation, fibrosis, ectopic tissue formation, degradation products and responses to repeated physiological loading. Manufacturing reproducibility, sterilization, storage stability, surgical delivery and compatibility with existing clinical procedures must also be addressed. Until these requirements are met, biomaterial and tissue-engineering systems should be regarded as promising preclinical platforms rather than established disease-modifying therapies.

**FIGURE 5:**
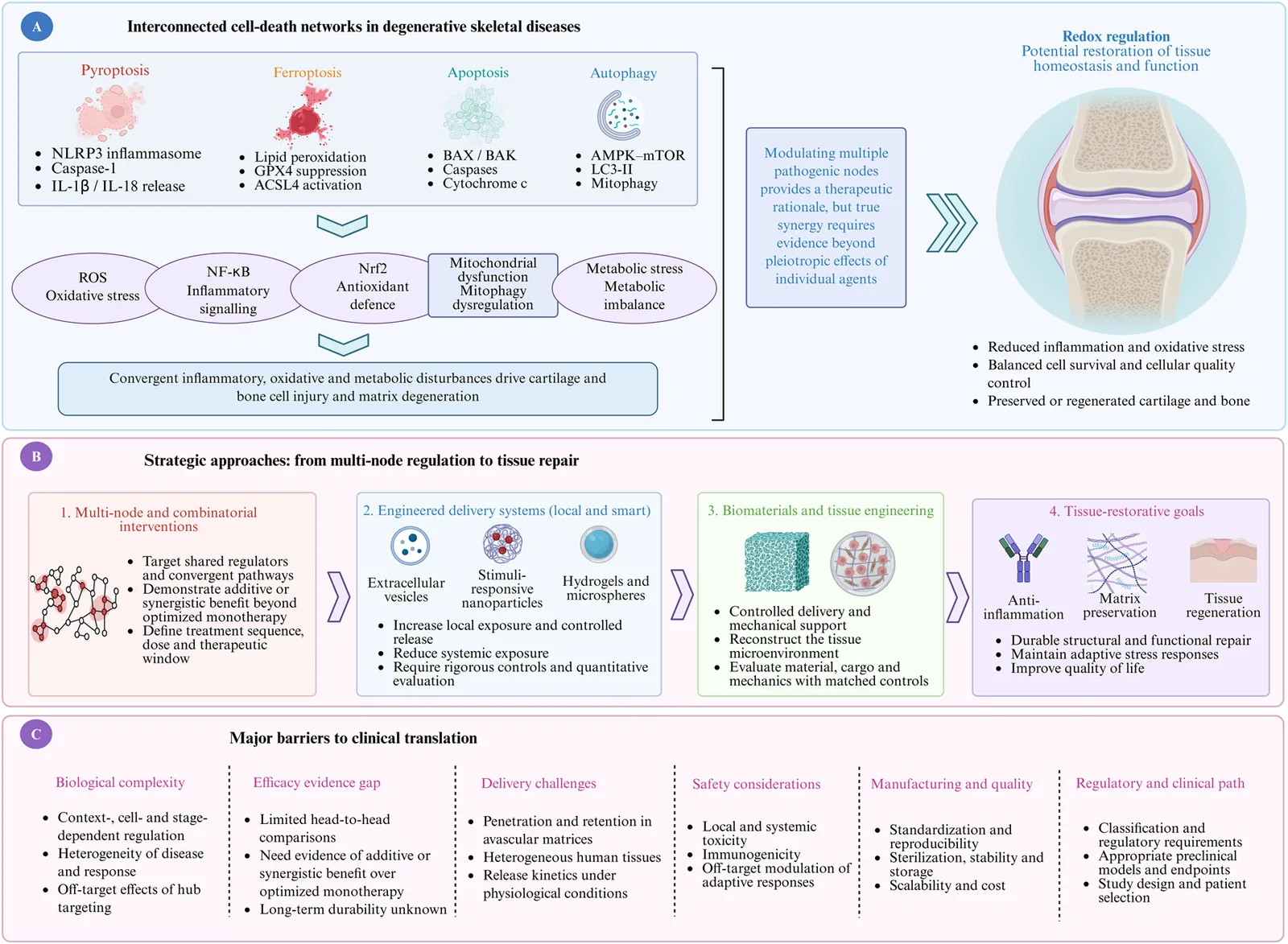
Therapeutic strategies and translational barriers for targeting cell-death-associated networks in degenerative skeletal diseases. **(A)** Apoptosis, pyroptosis, ferroptosis and autophagy-associated cell-fate regulation are influenced by convergent inflammatory, oxidative, mitochondrial and metabolic disturbances. Modulation of shared regulatory nodes may reduce cell injury and promote tissue homeostasis, but simultaneous effects on multiple pathways do not alone demonstrate therapeutic synergy. **(B)** Major therapeutic strategies include multi-node or combinatorial interventions, extracellular-vesicle- and stimuli-responsive delivery systems, biomaterials and tissue-engineering approaches, with the ultimate goals of controlling inflammation, preserving matrix integrity and restoring tissue structure and function. **(C)** Clinical translation remains limited by disease and cell-type heterogeneity, insufficient comparative efficacy evidence, delivery and retention challenges, local and systemic safety concerns, manufacturing variability and regulatory requirements. Abbreviations: ACSL4, acyl-CoA synthetase long-chain family member 4; AMPK, AMP-activated protein kinase; BAK, BCL-2 antagonist/killer; BAX, BCL-2-associated X protein; GPX4, glutathione peroxidase 4; GSDMD, gasdermin D; IL, interleukin; LC3, microtubule-associated protein 1 light chain 3; mTOR, mechanistic target of rapamycin; NF-κB, nuclear factor-κB; NLRP3, NLR family pyrin domain containing 3; NRF2, nuclear factor erythroid 2-related factor 2; ROS, reactive oxygen species.

Taken together, multi-node interventions, engineered delivery platforms and biomaterials provide complementary approaches to modulating cell-death-associated networks. Their translational value will depend not on the number of pathways or markers affected, but on demonstrated target dependency, superiority over simpler interventions, durable tissue-level benefit and acceptable safety, manufacturing and regulatory profiles. Representative preclinical multi-node interventions, engineered delivery systems and biomaterial platforms targeting cell-death-associated pathways, together with their principal translational limitations, are summarized in Table 3. As summarized in Figure 5, therapeutic modulation of cell-death-associated networks requires integration of multi-node interventions, engineered delivery systems and tissue-restorative approaches, while addressing biological, delivery, safety, manufacturing and regulatory barriers to clinical translation.

**TABLE 3:** Representative preclinical delivery and multi-node intervention platforms targeting cell-death-associated pathways.

Disease	Platform/intervention	Principal cargo or target	Cell-death-associated outcome	Preclinical delivery context	Mechanistic interpretation	Principal translational limitation	Ref.
osteoarthritis (OA)	Kartogenin-loaded M2-macrophage-membrane biomimetic liposomes	Kartogenin; macrophage-membrane components; cartilage-catabolic pathways	Reduced ferroptosis-associated chondrocyte injury and enhanced chondrogenic differentiation	Biomimetic intra-articular nanoplatform; cell and animal evaluation	Combines immunomodulatory, matrix-protective and ferroptosis-associated effects	Targeting advantage, membrane-source consistency, immunogenicity, biodistribution and superiority over free kartogenin require direct comparison	(Yue et al., 2025)
intervertebral disc degeneration (IVDD)	mesenchymal stromal cell (MSC)-derived extracellular-vesicle intervention	sequestosome 1 (p62)–Kelch-like ECH-associated protein 1(KEAP1)–nuclear factor erythroid 2-related factor 2(NRF2); glutathione peroxidase 4 (GPX4); acyl-CoA synthetase long-chain family member 4 (ACSL4)	Reduced oxidative-stress-induced nucleus pulposus (NP)-cell ferroptosis	Local disc delivery; preclinical	Vesicle-associated signalling activates antioxidant and lipid-peroxidation defence	extracellular vesicle (EV) heterogeneity, potency assays, dose, retention and comparison with free cargo and non-vesicle controls remain unresolved	(Chen et al., 2025d)
IVDD	Polydopamine nanoparticles	reactive oxygen species (ROS); iron handling; GPX4-associated antioxidant defence	Reduced NP-cell ferroptosis-associated injury	Local nanoparticle injection; preclinical	ROS scavenging and iron regulation reduce lipid-peroxidation stress	Long-term particle persistence, degradation, off-target metal binding, dose and large-animal disc penetration require evaluation	(Yang et al., 2023b)
IVDD	Extracellular-iron-adsorbing F127-diacrylate/tannic-acid hydrogel (FDA–TA)	Extracellular iron; ferritin heavy chain 1(FTH1); nuclear receptor coactivator 4(NCOA4); phosphoinositide 3-kinase (PI3K)–protein kinase B (AKT)	Reduced ferroptosis- and ferritinophagy-associated injury	Injectable hydrogel; preclinical	Combines extracellular iron adsorption, stabilization of intracellular iron handling and mechanical support	Component-specific effects, long-term durability, degradation, biocompatibility and performance in large-animal discs require testing	(Xu et al., 2024)
IVDD/cartilage endplate	CAP–NRF2 engineered extracellular vesicles	NRF2; dynamin-related protein 1 (DRP1); haem oxygenase 1(HO-1); NAD(P)H quinone dehydrogenase 1(NQO1); superoxide dismutase 2(SOD2)	Reduced mitochondrial-apoptosis-associated injury	Local engineered-EV delivery; preclinical	Cargo delivery supports antioxidant defence and mitochondrial dynamics	Receptor-dependent targeting, loading consistency, off-target uptake, immunogenicity and scalable manufacturing remain unproven	(Lin et al., 2024c)
OA	ROS-responsive NP@PolyRHAPM nanoplatform	Astaxanthin; rapamycin; ROS-responsive thioketal-containing polymer	Reduced inflammation- and apoptosis-associated chondrocyte injury	Intra-articular stimuli-responsive nanotherapy; preclinical	ROS scavenging, autophagy modulation and macrophage-state regulation	Release kinetics in heterogeneous joints, nanoparticle clearance, empty-carrier controls and durable efficacy require validation	(Li et al., 2024d)
OA	small interfering RNA targeting matrix metalloproteinase 13 (siMMP13)-loaded liposomes in an antioxidant injectable hydrogel	siMMP13; ROS; apoptosis-associated signalling	Reduced oxidative-stress-associated apoptosis and extracellular matrix (ECM) loss	Intra-articular composite hydrogel; preclinical	Combines gene silencing, ROS modulation and local retention	ribonucleic acid (RNA) stability, off-target silencing, dosing frequency, carrier toxicity and comparison with individual components are required	(Ji et al., 2024)
OA	Anti- transient receptor potential vanilloid 1 (TRPV1) gold nanorods with near-infrared irradiation	TRPV1; GPX4; NCOA4; lipid-peroxidation markers	Reduced ferroptosis-associated chondrocyte injury	Intra-articular photothermal intervention; preclinical	TRPV1-directed photothermal modulation is associated with reduced lipid peroxidation	Heat distribution, cartilage thermal safety, antibody orientation, gold persistence and procedural feasibility require assessment	(Li et al., 2024e)
OA	RAPA@MPB–matrix metalloproteinase 9 (MMP9) functionalized nanoplatform	Rapamycin; mechanistic target of rapamycin complex 1 (mTORC1); MMP9; cyclic GMP–AMP synthase (cGAS)–stimulator of interferon genes (STING); ROS	Reduced synovial-macrophage pyroptosis-associated inflammation	Intra-articular nanoparticle delivery; preclinical	ROS modulation and mitophagy-associated immunoregulation	Targeting requires quantitative comparator data; rapamycin-related immunosuppression, dose and chronic safety remain concerns	(Qi et al., 2024)
IVDD	dECM@exo thermosensitive extracellular-matrix hydrogel	Extracellular vesicles; NLR family pyrin domain containing 3(NLRP3)–caspase-1(CASP1)–gasdermin D (GSDMD); matrix metalloproteinase 13 (MMP13)	Reduced NP-cell pyroptosis-associated injury	Local injectable hydrogel with sustained EV release; preclinical	Prolonged local retention with inflammatory and matrix regulation	Hydrogel and EV effects must be separated; source variability, sterilization, mechanical durability and surgical delivery require study	(Xing et al., 2021)
Diabetic osteoporosis (OP)	Curcumin-loaded deoxyribonucleic acid (DNA) tetrahedron nanosystem	Curcumin; NRF2–GPX4 antioxidant pathway	Reduced ferroptosis-associated injury and enhanced osteogenic differentiation	Systemic or local nanodelivery in preclinical diabetic-OP models	Cargo delivery modifies antioxidant defence and osteogenesis	DNA-nanostructure stability, biodistribution, immune recognition, pharmacokinetics and superiority over free curcumin remain unresolved	(Li et al., 2024f)
IVDD	PVA–tsPBA@SLC7A11 modified-RNA hydrogel	solute carrier family 7 member 11(SLC7A11) modified RNA; ROS	Reduced NP-cell ferroptosis	ROS-responsive local injectable hydrogel; preclinical	Stimulus-responsive RNA release restores cystine transport and antioxidant defence	RNA-expression duration, release reproducibility, immunogenicity, off-target expression and repeated-dose safety require evaluation	(Gao et al., 2024c)
OA	Dihydromyricetin-loaded hyaluronic acid methacrylate (HAMA)–gelatin methacryloyl (GelMA)–PBA microspheres	Dihydromyricetin; sirtuin 3(SIRT3); ROS; mitochondrial quality control	Reduced mitochondrial-apoptosis-associated chondrocyte injury	Dual-responsive intra-articular microspheres; preclinical	Mitophagy-associated mitochondrial protection with sustained local release	Independent effects of dihydromyricetin, microsphere mechanics and release chemistry require matched controls	(XIA et al., 2023b)
OA/subtalar joint	Platelet-rich-plasma extracellular vesicles in thermosensitive Exo-Gel	platelet-rich plasma (PRP)-derived EV cargo; transforming growth factor beta 1 (TGF-β1)- and stromal cell-derived factor 1(SDF-1)-associated reparative signalling	Reduced inflammation- and apoptosis-associated cartilage injury	Local injectable thermosensitive gel; preclinical	Prolonged local retention with reparative and anti-inflammatory signalling	PRP donor variability, EV definition, growth-factor safety, mechanical performance and relevance beyond the specific joint model require validation	(Zhang et al., 2022b)
OA	CAP-Lipo@KGN@Mg-ZIF hybrid platform	Kartogenin; magnesium ion (Mg2+); zinc ion (Zn2+); immune and osteochondral-remodelling pathways	Reduced chondrocyte apoptosis and matrix catabolism with remodelling effects	Single intra-articular administration in preclinical models	Combines cartilage protection, immune regulation and osteochondral remodelling	True synergy requires optimized single-component controls; metal-ion release, particle degradation, long-term toxicity and manufacturing complexity remain barriers	(Shao et al., 2025)
OA	MI@UN macrophage-membrane biomimetic nanoparticles	interleukin-10(IL-10)-associated macrophage regulation; HIF-1α(HIF-1α); GPX4; ACSL4	Reduced ferroptosis-associated chondrocyte injury	Intra-articular biomimetic nanodelivery; preclinical	Macrophage-metabolism regulation is combined with ferroptosis-associated cartilage protection	Cell-membrane sourcing, batch reproducibility, receptor specificity, immune effects and biodistribution require direct testing	(Li et al., 2025c)
IVDD	HACS@CPP- microRNA (miR)-Exo dynamic ECM-mimetic hydrogel	miR-221–3p; interferon regulatory factor 8(IRF8)–signal transducer and activator of transcription 1(STAT1); SLC7A11–GPX4	Reduced NP-cell ferroptosis	Postoperative local filling of the NP cavity; preclinical	Sustained EV release and regulation of an inflammatory–ferroptotic axis	Surgical applicability, annular integrity, leakage, disc mechanics, EV dose and long-term retention remain uncertain	(Wang W. et al., 2025)
IVDD	PGA-Cu@SS08 nanoparticle-composite hydrogel	NRF2-associated antioxidant defence; iron handling; mitochondrial function	Reduced NP-cell ferroptosis-associated injury	Local injectable nanocomposite hydrogel; preclinical	ROS scavenging, iron regulation and mitochondrial protection	Copper-related toxicity, redox overcorrection, hydrogel degradation, local retention and large-animal safety require assessment	(Guo et al., 2025)
OA	SCT@AHAMA penetrative micro/nanohydrogel microspheres	ROS; mitochondrial function; MMP13; collagen type II alpha 1 chain (COL2A1); platelet-derived growth factor-BB (PDGF-BB)	Reduced oxidative-stress-associated chondrocyte apoptosis	Intra-articular microsphere delivery; preclinical	Cartilage penetration with regulation of cartilage and subchondral-bone metabolism	Penetration claims require quantitative depth comparison; PDGF-BB dose, subchondral effects, clearance and repeated-loading durability require study	(Zuo et al., 2024)
OP	Sodium butyrate	phosphatase and tensin homolog (PTEN)–PI3K–AKT; GPX4-associated antioxidant defence	Reduced ferroptosis-associated lipid peroxidation in osteogenic cells	Systemic administration in ovariectomy (OVX) mice with in vitro bone marrow mesenchymal stromal cell (BMSC) validation	Metabolite-associated PTEN regulation supports AKT signalling and osteogenic differentiation	Systemic pleiotropy, microbiome dependence, exposure levels, human pharmacology and specificity for ferroptosis remain unresolved	(Li et al., 2025d)

Abbreviations: ACSL4, acyl-CoA, synthetase long-chain family member 4; AKT, protein kinase B; BMSC, bone marrow mesenchymal stromal cell; CASP1, caspase-1; cGAS, cyclic GMP–AMP, synthase; COL2A1, collagen type II, alpha 1 chain; DNA, deoxyribonucleic acid; DRP1, dynamin-related protein 1; ECM, extracellular matrix; EV, extracellular vesicle; FTH1, ferritin heavy chain 1; GelMA, gelatin methacryloyl; GPX4, glutathione peroxidase 4; GSDMD, gasdermin D; HAMA, hyaluronic acid methacrylate; HIF-1α, hypoxia-inducible factor 1 alpha; HO-1, haem oxygenase 1; IL-10, interleukin-10; IRF8, interferon regulatory factor 8; IVDD, intervertebral disc degeneration; KEAP1, Kelch-like ECH-associated protein 1; Mg^2+^, magnesium ion; miR, microRNA; MMP9, matrix metalloproteinase 9; MMP13, matrix metalloproteinase 13; MSC, mesenchymal stromal cell; mTORC1, mechanistic target of rapamycin complex 1; NCOA4, nuclear receptor coactivator 4; NLRP3, NLR, family pyrin domain containing 3; NP, nucleus pulposus; NQO1, NAD(P)H quinone dehydrogenase 1; NRF2, nuclear factor erythroid 2-related factor 2; OA, osteoarthritis; OP, osteoporosis; OVX, ovariectomy; p62, sequestosome 1; PDGF-BB, platelet-derived growth factor-BB; PI3K, phosphoinositide 3-kinase; PRP, platelet-rich plasma; PTEN, phosphatase and tensin homolog; RNA, ribonucleic acid; ROS, reactive oxygen species; SDF-1, stromal cell-derived factor 1; siMMP13, small interfering RNA, targeting matrix metalloproteinase 13; SIRT3, sirtuin 3; SLC7A11, solute carrier family 7 member 11; SOD2, superoxide dismutase 2; STAT1, signal transducer and activator of transcription 1; STING, stimulator of interferon genes; TGF-β1, transforming growth factor beta 1; TRPV1, transient receptor potential vanilloid 1; Zn^2+^, zinc ion.

## 7 Discussion

Substantial progress has been made in defining the involvement of PCD in degenerative skeletal diseases, but the current evidence remains incomplete at both mechanistic and translational levels. Most studies examine individual death-associated pathways under simplified experimental conditions, whereas their temporal hierarchy, causal interdependence and cell-specific consequences remain insufficiently resolved. In particular, the same death programme may function as an adaptive response, an injury amplifier or a terminal execution mechanism depending on the affected cell population, disease stage and local microenvironment. The distribution of evidence is also uneven across diseases and anatomical compartments. OA research is dominated by articular chondrocyte models, with comparatively less mechanistic evidence from the synovium and subchondral bone. IVDD studies predominantly focus on nucleus pulposus cells, whereas annulus fibrosus and cartilage endplate cells remain less comprehensively characterized. In OP, evidence is distributed among osteoblasts, osteoclasts, osteocytes and BMSCs, but the tissue-level consequence of pathway modulation differs markedly among these lineages. For example, inhibition of ferroptosis may preserve osteoblast or osteocyte viability and support bone formation, whereas suppression of osteoclast ferroptosis may prolong osteoclast survival and enhance bone resorption. Mechanisms identified in one cell type or anatomical niche should therefore not be extrapolated automatically to another disease context, even when similar upstream regulators are involved.

Interpretation is further complicated by the limited specificity of many commonly used molecular readouts. Reduced GPX4 or SLC7A11 expression, increased lipid ROS and altered mitochondrial morphology are consistent with ferroptosis-associated injury but do not independently establish iron-dependent cell death. Stronger evidence requires demonstration of iron and lipid-peroxidation dependency, together with rescue by mechanistically distinct ferroptosis-targeted interventions or genetic perturbation. Similarly, increased NLRP3, caspase-1 or gasdermin cleavage may indicate inflammasome activation without demonstrating complete pyroptotic execution. Membrane permeabilization, lytic cell death and pathway-specific dependency should therefore be assessed alongside molecular markers. Concurrent detection of apoptotic, pyroptotic and necroptotic proteins likewise supports multimodal PCD activation but should not be considered definitive PANoptosis unless integrated execution, causal dependency or PANoptosome-related molecular assembly is demonstrated. Autophagy-associated findings require comparable conceptual caution. Increased LC3-II may reflect enhanced autophagosome formation or impaired lysosomal degradation, and static measurements cannot distinguish these possibilities. Dynamic assessment of autophagic flux is therefore essential. Moreover, different selective autophagy programmes may have opposing effects: mitophagy can protect cells by eliminating dysfunctional mitochondria and limiting ROS accumulation, whereas NCOA4-mediated ferritinophagy can increase the labile iron pool and promote ferroptosis. Autophagy should therefore be understood as a context-dependent regulator of cellular adaptation and death susceptibility rather than as a uniformly protective process or an equivalent PCD execution pathway. Evidence of altered autophagy-associated markers alone is also insufficient to establish autophagy-dependent cell death.

Pharmacological studies represent another important source of uncertainty. Many compounds described as modulators of a particular death pathway also exert antioxidant, anti-inflammatory, metabolic or mitochondrial effects. Simultaneous reductions in several death-associated markers may therefore reflect attenuation of a shared upstream stressor rather than direct inhibition of each execution pathway or genuine therapeutic synergy. Establishing target specificity will require genetic perturbation, orthogonal pharmacological tools, pathway-specific rescue experiments and explicit evaluation of off-target effects. Similarly, a single pleiotropic compound should not be regarded as a combinatorial therapy unless its independent mechanistic components and additive or synergistic effects have been demonstrated against appropriate single-target comparators. The limitations of experimental models also constrain translation. Destabilization of the medial meniscus (DMM), puncture-induced IVDD and ovariectomy (OVX) models reproduce selected structural and molecular features but do not fully capture the chronicity, heterogeneity and multimorbidity of human disease. Preclinical studies frequently use young animals, short observation periods and preventive treatment protocols, whereas patients commonly present at an older age with established structural damage, metabolic comorbidities and previous treatment exposure. Cellular experiments may additionally rely on supraphysiological concentrations of cytokines, hydrogen peroxide, iron or other stressors. These differences can alter the dominant death response and therapeutic sensitivity, limiting direct extrapolation to human disease.

Emerging multi-omics, single-cell and spatial technologies provide opportunities to address several of these limitations. Integrated genomic, transcriptomic, proteomic and metabolomic analyses can identify regulatory nodes shared across inflammatory, metabolic and death-associated pathways (Wang C. et al., 2025; Lu et al., 2026). Single-cell and spatial approaches can further resolve which cell populations activate particular programmes and how these responses vary across anatomical niches and disease stages. Artificial intelligence and machine-learning methods may assist in integrating heterogeneous datasets and prioritizing candidate regulators or therapeutic combinations (Zhou et al., 2023). However, computational associations do not establish causal relationships, and their utility remains dependent on dataset quality, model generalizability, biological interpretability and experimental validation.

Future research should therefore proceed along complementary mechanistic and translational axes. Mechanistically, priority should be given to longitudinal and lineage-specific studies capable of distinguishing adaptive stress responses, shared upstream regulation, sequential transitions and direct molecular interdependence. Live-cell monitoring, spatially resolved profiling, inducible genetic models and time-controlled pathway perturbation will be particularly important for defining when and where cell-fate transitions occur. Disease-specific investigation is equally necessary: OA studies should distinguish cartilage, synovial and subchondral-bone responses; IVDD studies should extend beyond nucleus pulposus cells to annulus fibrosus and cartilage endplate compartments; and OP studies should evaluate osteoblasts, osteocytes, osteoclasts and BMSCs separately. Translationally, the immediate priority should not be indiscriminate simultaneous inhibition of multiple death programmes. Instead, causal regulatory hubs should first be validated in human-relevant tissues, organoids, *ex vivo* systems and appropriately aged or comorbid animal models. Multi-target combinations should then be compared directly with optimized monotherapies to determine whether they provide additive or synergistic benefit, whether the effect is durable and whether adaptive or reparative responses are preserved. Targeted delivery and biomaterial systems should likewise be evaluated according to quantitative biodistribution, target engagement, therapeutic window, repeat-dose safety, immunogenicity, degradation, manufacturing reproducibility and compatibility with clinical practice. Treatment design should ultimately be matched to the dominant cell population, anatomical compartment, disease stage and local microenvironment rather than transferred directly between OA, IVDD and OP.

The central conclusion of this Review is therefore not that a single death modality uniformly dominates degenerative skeletal diseases, nor that the simultaneous detection of several death-associated markers necessarily indicates molecular crosstalk. PCD in OA, IVDD and OP is more appropriately understood as an evidence-graded and context-dependent network shaped by cell identity, anatomical niche, disease stage and local inflammatory, redox, metabolic and mechanical stress. Shared upstream regulators provide a rationale for cross-disease comparison, but they do not establish equivalent downstream functions or therapeutic responses. Most current evidence supports functional coexistence or regulation by shared upstream signals, whereas direct molecular conversion, sequential dependency and integrated execution remain less extensively demonstrated. Distinguishing these levels of evidence provides a more rigorous foundation for translating cell-death biology into context-specific and potentially disease-modifying interventions for degenerative skeletal disorders.

## Glossary

ACSL4: acyl-CoA synthetase long-chain family member 4
ADAMTS5: a disintegrin and metalloproteinase with thrombospondin motifs 5
AMPK: AMP-activated protein kinase
Bax: Bcl-2-associated X protein
BMSCs: bone marrow mesenchymal stem cells
c-Fos: cellular Fos proto-oncogene
DMM: destabilization of the medial meniscus
DNMT: DNA methyltransferase
ECM: extracellular matrix
ER: endoplasmic reticulum
EZH2: enhancer of zeste homolog 2
FTH1: ferritin heavy chain 1
GBP5: guanylate-binding protein 5
GPX4: glutathione peroxidase 4
GSDMD: gasdermin D
GSDME: gasdermin E
HMGB1: high mobility group box 1
IRF1: interferon regulatory factor 1
JNK1: c-Jun N-terminal kinase 1
JUN: jun proto-oncogene, AP-1 transcription factor subunit
LC3: microtubule-associated protein 1 light chain 3
MAPK: mitogen-activated protein kinase
MLKL: mixed lineage kinase domain-like protein
MMP: matrix metalloproteinase
mTOR: mechanistic target of rapamycin
NCOA4: nuclear receptor coactivator 4
NOX: NADPH oxidase
NP: nucleus pulposus
NT3: neurotrophin-3
OA: osteoarthritis
OP: osteoporosis
OVX: ovariectomy
PANoptosis: pyroptosis, apoptosis and necroptosis
PCD: Programmed cell death
PINK1: PTEN-induced putative kinase 1
PTEN: phosphatase and tensin homolog
RANKL: receptor activator of nuclear factor kappa-B ligand
RIPK1: receptor-interacting serine/threonine-protein kinase 1
RIPK3: receptor-interacting serine/threonine-protein kinase 3
ROS: reactive oxygen species
SLC7A11: solute carrier family 7 member 11
SOD: superoxide dismutase
STING: stimulator of interferon genes
TAK1: transforming growth factor-β-activated kinase 1
TLR4: toll-like receptor 4
TrkC: tropomyosin receptor kinase C
TXNIP: thioredoxin-interacting protein
